# A yeast mating platform for multiplex screening of fungal GPCR–ligand interactions

**DOI:** 10.1073/pnas.2521198122

**Published:** 2025-10-24

**Authors:** Giovanni Schiesaro, Melani Mariscal, Mathias Jönsson, Ricardo Tenente, Mathies Brinks Sørensen, Marcus Wäneskog, María Victoria Aguilar-Pontes, Agustina Undabarrena, Marcus Deichmann, Emma E. Hoch-Schneider, Viji Kandasamy, Thomas M. Frimurer, Antonio Di Pietro, Line Katrine Harder Clemmensen, Michael Krogh Jensen, Emil Damgaard Jensen

**Affiliations:** ^a^Novo Nordisk Foundation Center for Biosustainability, Technical University of Denmark, Kongens Lyngby 2800, Denmark; ^b^Departamento de Genética, Campus de Excelencia Internacional Agroalimentario, Universidad de Córdoba, Córdoba 14014, Spain; ^c^Novo Nordisk Foundation Center for Basic Metabolic Research, Faculty of Health and Medical Sciences, University of Copenhagen, Copenhagen DL-2200, Denmark; ^d^Department of Applied Mathematics and Computer Science, Technical University of Denmark, Kongens Lyngby 2800, Denmark

**Keywords:** cell–cell communication, fungal GPCRs, high-throughput screening, consortia, plant pathogen

## Abstract

Fungi are essential members across ecosystems, yet phytopathogenic fungi pose an increasing risk to crop yields. Despite their ecologic importance, cell–cell communication in fungi is underexplored, partly due to the lack of high-throughput techniques. Here, we developed a Yeast Mating Platform (YeMaP) to investigate the interaction between fungal G protein–coupled receptors (GPCRs) and pheromone peptides. We used YeMaP for high-throughput screening of 8,000 pheromone sequences and identified peptides with improved agonism or antagonism action. We found that these peptides can be applied in a native fungal system such as the plant pathogen *Fusarium oxysporum*, to control hyphal chemotropism and reduce plant root penetration. Additionally, we utilized YeMaP in a one-pot assay to investigate how abiotic factors influence the communication of multiple pheromone–GPCR combinations and found that the cell–cell communication mediated by the GPCR Ste2 from *F. oxysporum* signaled robustly across different abiotic factors, while other fungal GPCR–pheromone interactions were more sensitive to changes. Taken together, YeMaP accelerates the identification of fungal GPCR–peptide interactions by enabling one-pot assays, and serves as a model system for studying fungal cell–cell communication.

## Significance

Fungal pathogens rely on G protein–coupled receptors (GPCRs) to sense environmental cues and coordinate host infection. By establishing a yeast mating platform for multiplex GPCR–ligand screening, we identify agonist and antagonist peptides that can interfere with fungal cell–cell communication. This work not only accelerates the study of fungal GPCR–ligand interactions but also demonstrates, for the phytopathogen *Fusarium oxysporum*, that interfering with GPCR-mediated cell–cell communication is a promising target for antifungal strategies in agriculture.

Fungi are indispensable for fundamental processes of global ecosystems such as the cycling of carbon, nitrogen, and phosphorus, all of which are critical for the survival of plants and animals (1). At the same time, phytopathogenic fungi pose an increasing threat to global food security (2). As climate change intensifies, the available data and simulation models indicate that plant disease pressures are likely to rise substantially in the near future (3).

To address these emerging challenges, we need a greater understanding of how intra- and interspecies communication occurs in fungal consortia. G protein–coupled receptors (GPCRs) are master switches that convert external stimuli into a coordinated cellular response (4). Through GPCRs, fungi can detect a variety of chemical signals including pheromones, nutrients, ions, hydrophobic surfaces, or light, allowing them to regulate their development, metabolism, and virulence (5). However, our current understanding of the role of fungal GPCRs is limited (5), and interactions between GPCRs and potential ligands are often poorly understood, thus hindering the development of new antifungal drugs and sustainable pest management systems for agriculture (6, 7).

The model yeast *Saccharomyces cerevisiae* encodes three GPCRs, two of which are mating receptors, *STE2* and *STE3* expressed in *MAT***a** and *MAT*α cells, respectively (8). To form diploid cells, haploid *MAT*α cells communicate through an unmodified short peptide (α-factor) to *MAT***a** cells, which respond with a posttranslationally modified peptide (**a**-factor) (9). In contrast, in the asexual plant pathogen *Fusarium oxysporum* (*Fo*), autocrine pheromone communication regulates spore germination (10), and the *Fo STE2* homolog is required for hyphal chemotropism toward plant roots (11). Similar results have recently been reported in *Fusarium graminearum* (12), pointing toward a conserved mechanism of plant recognition across the ascomycota phylum. Fungal mating receptors have been expressed in *S. cerevisiae* for pathogen detection (13) or to establish orthogonal communication (14) in biosensor strains. However, cell–cell communication between GPCRs and pheromones has never been explored in multiplex assays to mimic the presence of multiple fungal organisms, although such a setup would better reflect the complexity of real-world interactions (15). Additionally, fungal pheromones are partly conserved among closely related species (16) and thus could function in interspecies communication (17). This suggests that the yeast mating system could serve as a tool to study fungal cell–cell communication.

Unlike GPCR-based biosensors (18), successful yeast mating requires the secretion and detection of pheromones along with the downstream activation of a developmental program for cell and nuclear fusion (19). Here, we established a yeast mating platform (YeMaP) for analyzing interactions between fungal GPCRs and pheromone variants. We used YeMaP to study fungal cell–cell communication from four different perspectives: i) the interaction between a fungal GPCR and a pheromone, ii) screening of a library of pheromone variants for identification of a synthetic peptide with higher potency toward the *F. oxysporum* Ste2 GPCR, iii) modeling cell–cell communication within fungal consortia, and iv) assessing the effects of external factors such as pH, nitrogen source, plant peroxidases, or different pheromone supplementations, on a network of library-on-library interactions. We further validate that both natural and artificial pheromones selected from YeMaP screening also confer function in the native fungal pathogen *F. oxysporum,* demonstrating that they trigger strong hyphal chemotropism while interfering with fungal penetration of plant roots. Altogether, YeMaP accelerates functional analysis of isolated fungal GPCRs, enabling large-scale exploration of GPCR-targeted pest management strategies.

## Results

### Engineering Yeast Mating to Study Fungal GPCR–Pheromone Interactions (One-on-One).

**Fig. 1.**
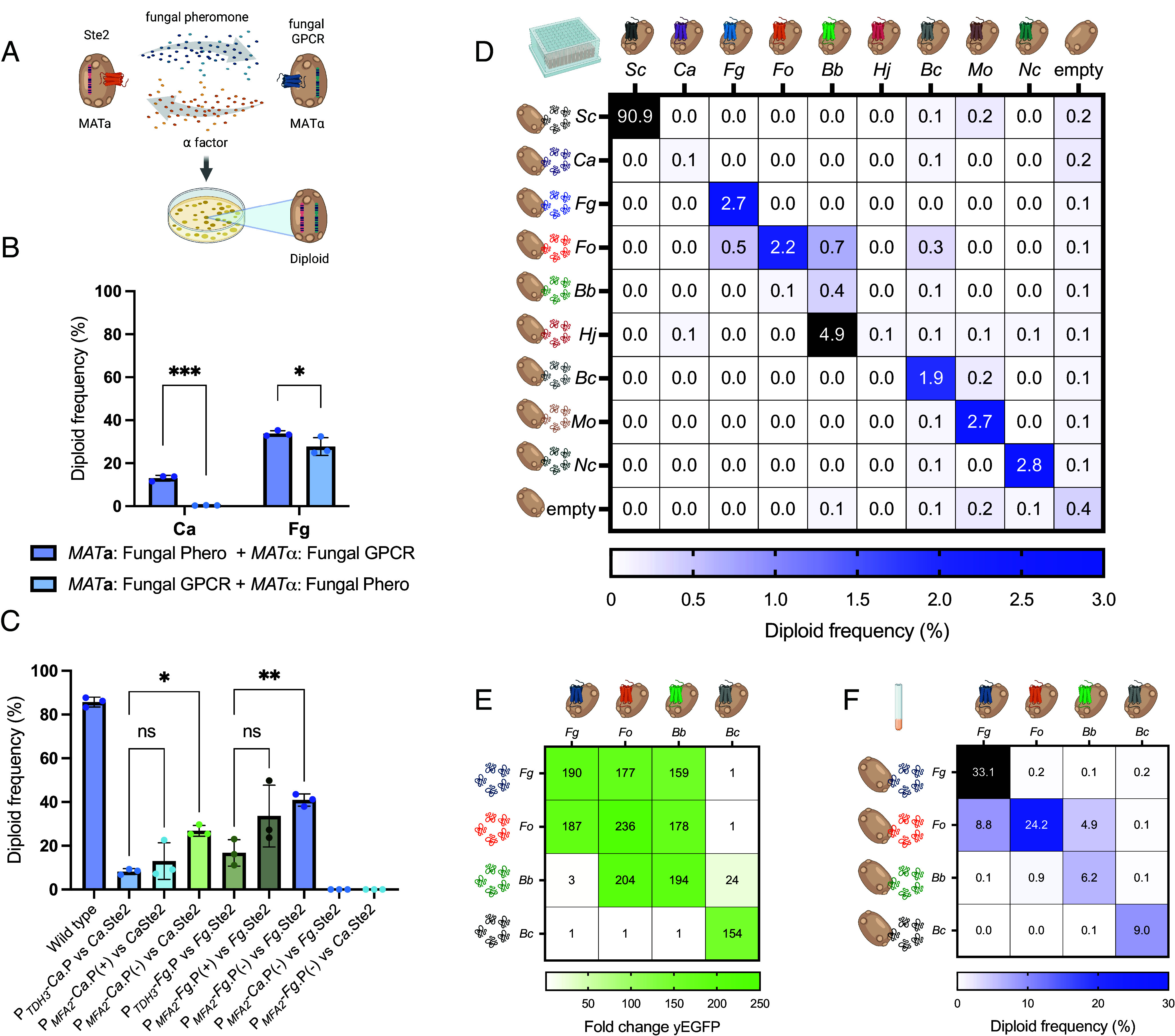
YeMaP correctly identifies fungal GPCR–pheromone interactions. (*A*) The One-on-One setting involves coculturing two yeast strains in which half of the mating communication system has been replaced with heterologous fungal components; one cell expresses a given fungal Ste2 GPCR homolog while a second cell secretes a given fungal alpha pheromone. After successful mating, diploid cells are formed and selected. (*B*) Comparison of cocultures expressing *C. albicans* (*Ca*) or *F. graminearum* (*Fg*) components under the following conditions: *MAT***a** cells expressing either the *Ca* or *Fg* alpha pheromone from the *MFA2* promoter (*Ca*: GEN29, *Fg*: GEN35) and *MAT*α cells expressing either the *Ca* or *Fg* Ste2 GPCR (*Ca*: GEN36, *Fg*: GEN37); or *MAT***a** cells expressing the fungal GPCR (*Ca*: GEN100, *Fg*: GEN78) and *MAT*α cells expressing the *Ca* or *Fg* alpha pheromone from the *MFα1* promoter (*Ca*: GEN76, *Fg*: GEN77). *MFA2* and *MFα1* promoters were selected as the strongest inducible pheromone promoters based on previous studies (20, 25). (*C*) Diploid frequency comparison between different designs. From left to right: GEN18 and GEN27 were cocultured as a positive control (Wild type); GEN36 (*Ca*.Ste2) cocultured with GEN28 (P*_TDH3_*-*Ca*.P); with GEN29 (P*_MFA2_*-*Ca*.P); or with GEN55 (P*_MFA2_*-*Ca*.P, *mfa1*Δ, *mfa2*Δ). GEN37 (*Fg*.Ste2) cocultured with GEN34 (P*_TDH3_*-*Fg*.P); with GEN35 (P*_MFA2_*-*Fg*.P); or with GEN58 (P*_MFA2_*-*Fg*.P, *mfa1*Δ, *mfa2*Δ); and GPCRs combined with noncognate pheromone GEN55 + GEN37; and GEN58 + GEN36. (*D*) Yeast mating matrix with *MAT*α strains individually expressing eight different fungal Ste2 GPCRs (columns), cocultured with either of eight alpha pheromone-secreting *MAT***a** strains (rows). As positive and negative controls, respectively, strains expressing either *Sc* Ste2 or alpha pheromone, or strains lacking a GPCR or a pheromone were used. Blue gradient is set to a maximum of 3% of diploid frequency, with values >3% colored in black. Complete list of organisms and pheromone sequences is in *SI Appendix*, Table S1. (*E*) Activation levels of *Fg*.Ste2 (CPK366), *Fo*.Ste2 (GEN70), *Bb*.Ste2 (GEN71), and *Bc*.Ste2 (GEN128) biosensors (columns), incubated with 10 μM alpha pheromone from *Fg*, *Fo*, *Bb*, or *Bc* (rows). The fluorescent signal was normalized to the yEGFP background value (receptor without pheromone). (*F*) Validation of the mating matrix in culture tubes. Strains individually expressing the four indicated GPCRs (columns) were cocultured with those secreting the indicated alpha pheromone (rows). Blue gradient is set to a maximum of 30% of diploid frequency, with values >30% colored in black. Plating was performed in three technical triplicates in *D*. Cocultures were performed in three biological replicates and three technical replicates in *B*, *C*, and *F*. Experiments were conducted in four biological replicates in *E*. Statistical significance was determined in *B* through two-way ANOVA with Tukey’s multiple comparisons (*P ≤ 0.05, ***P ≤ 0.001); and in *C* with one-way ANOVA with Tukey’s multiple comparisons tests in GraphPad Prism (*P ≤ 0.05, **P ≤ 0.01).

To define the best design for establishing a synthetic YeMaP to study network interactions among multiple fungal GPCRs, we initially focused on the Ste2 GPCRs from *Candida albicans* (*Ca*) and *F. graminearum* (*Fg*), which had previously been expressed in yeast (14, 20, 21) (Fig. 1*A*). Since asymmetry in sexual pheromones is not required between yeast mating pairs (22), we first sought to identify which combination of mating-types was most suitable for heterologous GPCR expression and alpha pheromone secretion (23). To this aim, we cocultured two yeast strains of opposite mating-types expressing either a fungal GPCR or a cognate pheromone, followed by growth-based diploid selection on plates as a readout of successful mating (22, 24). We found that substitution of the native yeast *STE3* GPCR by a heterologous *STE2*-like GPCR in *MAT*α cells, combined with production of its cognate alpha pheromone in cocultivated *MAT***a** partner cells resulted in the highest diploid frequencies. Specifically, for the *Ca* GPCR–pheromone pair, this setup yielded a 33-fold higher diploid frequency than the opposite combination (*P* < 0.0001) (Fig. 1*B*), corroborating a previous study by Huberman et al. (24).

Next, we asked whether mimicking the induction of pheromone expression during native yeast mating is an essential feature (9, 20). This was done by comparing diploid frequencies obtained from strains showing either constitutive or inducible expression of the cognate heterologous pheromone. Higher diploid frequencies were obtained when the heterologous pheromones were expressed from the inducible *MFA2* promoter (26) compared to the strong constitutive *TDH3* promoter (27), although the difference was only statistically significant when the strains were deleted for both **a**-factor genes, *MFA1* and *MFA2* (*P* = 0.039 with *Ca*.Ste2 and *P* = 0.005 with *Fg*.Ste2) (Fig. 1*C*). Importantly, no diploid formation was observed when a given GPCR-expressing strain was cocultured with a strain expressing a noncognate pheromone (Fig. 1*C*).

Once the optimal strain design had been established, we next evaluated the specificity of the YeMaP by performing a “One-on-One” mating matrix with strains individually expressing eight different fungal GPCR and strains individually secreting eight different pheromones. The native *Sc* system and an empty construct were added as positive and negative controls, respectively (Fig. 1*D*). In general, the species-specific pheromone–GPCR combinations yielded the highest diploid frequencies, although we observed promiscuity of the *Beauveria bassiana* (*Bb*) GPCR (*Bb*.Ste2) toward pheromones from other fungi such as *F. oxysporum* (*Fo*) or *Hypocrea jecorina* (*Hj*). Furthermore, *Fo* pheromone induced diploid formation upon coculture with cells expressing the GPCRs *Fg*.Ste2, *Fo*.Ste2, or *Bb*.Ste2 (Fig. 1*D*).

We performed further validation in a yeast-based biosensor assay (14) with four selected GPCRs [*Fg*.Ste2, *Fo*.Ste2, *Bb*.Ste2, and *Botrytis cinerea* (*Bc*) *Bc*.Ste2] and their chemically synthesized cognate pheromones. Biosensor assays (14) were performed using a Green Fluorescent Protein (yEGFP) reporter as readout (Fig. 1*E*), while in parallel comparing the results to the mating-based selection assay in culture tubes, to evaluate which of the two approaches provided the highest resolution (Fig. 1*F*). We observed a binary (on/off) behavior for the biosensors, with *Bb*.Ste2 showing almost full activation in response to *Fg* pheromone, and *Fo*.Ste2 exhibiting a strong activation when exposed to either *Bb* or *Fg* pheromones (Fig. 1*E*). However, these three strong heterologous GPCR–ligand responses did not correlate with diploid formation rate in the YeMaP, which were lower in comparison to the corresponding cognate pheromone interactions (Fig. 1*F*). The observed differences between mating pathway activation in the biosensor assay and diploid formation in YeMaP suggests that pheromone-induced signaling alone is not sufficient to trigger the much more complex mating response. Validation of the mating matrix in culture tubes recapitulated the same trends as shown in Fig. 1*D*, although a higher diploid frequency was obtained in this condition (Fig. 1*F*).

To further explore on the observed cross-reactivity between *Bb*.Ste2 and *Fo* pheromone detected in the YeMaP assay, along with the documented protective effect of *Bb* against *Fo* on crops (28, 29), we investigated whether the species *Bb* is able to sense and respond to *Fo* pheromone. Indeed, either *Bb* or *Fo* pheromone induced a significant chemotropic response in *Bb* germ tubes (*P* < 0.05) at a concentration of 378 μM, whereas a scrambled version of the *Fo* pheromone did not (*SI Appendix*, Fig. S1).

Taken together, these results demonstrate that the YeMaP assay provides a superior resolution in deciphering GPCR–pheromone interactions than the previously reported yeast biosensor assay. We therefore asked whether YeMaP, unlike biosensors, is able to scale investigations by enabling mating-based selection of pheromone–GPCR interactions in a multiplex setup.

### Screening a Library of Pheromone Variants on a Fungal GPCR to Identify Improved Pheromone Agonists (Library-on-One).

We next asked whether the YeMaP assay can be used to screen for improved alpha pheromone agonists. Several *Sordariomycetes* with relevant ecological roles have alpha pheromones sharing a similar backbone (16, 30). Given this natural resemblance, and building on the deciphered interactions shown above (Fig. 1*F*), we constructed an alpha pheromone library (named YPL1) encoding 8,000 pheromone peptide variants to reach saturation of three internal residues in the sequence WCXXXGQPCW (where X is any possible amino acid in position 3, 4, and 5), while preserving the two essential residues Gly6-Gln7 required for maintaining the three-dimensional structure of the *Fo* alpha pheromone (31). By adapting a recently established state-of-the-art yeast transformation method (32), we transformed over 1.8 × 10^6^ yeast cells, obtaining a 6.9-fold coverage of our DNA-encoded pheromone library (8,000 peptides = 262,144 nucleotide variants), with each individual cell expressing a single alpha pheromone variant.

**Fig. 2.**
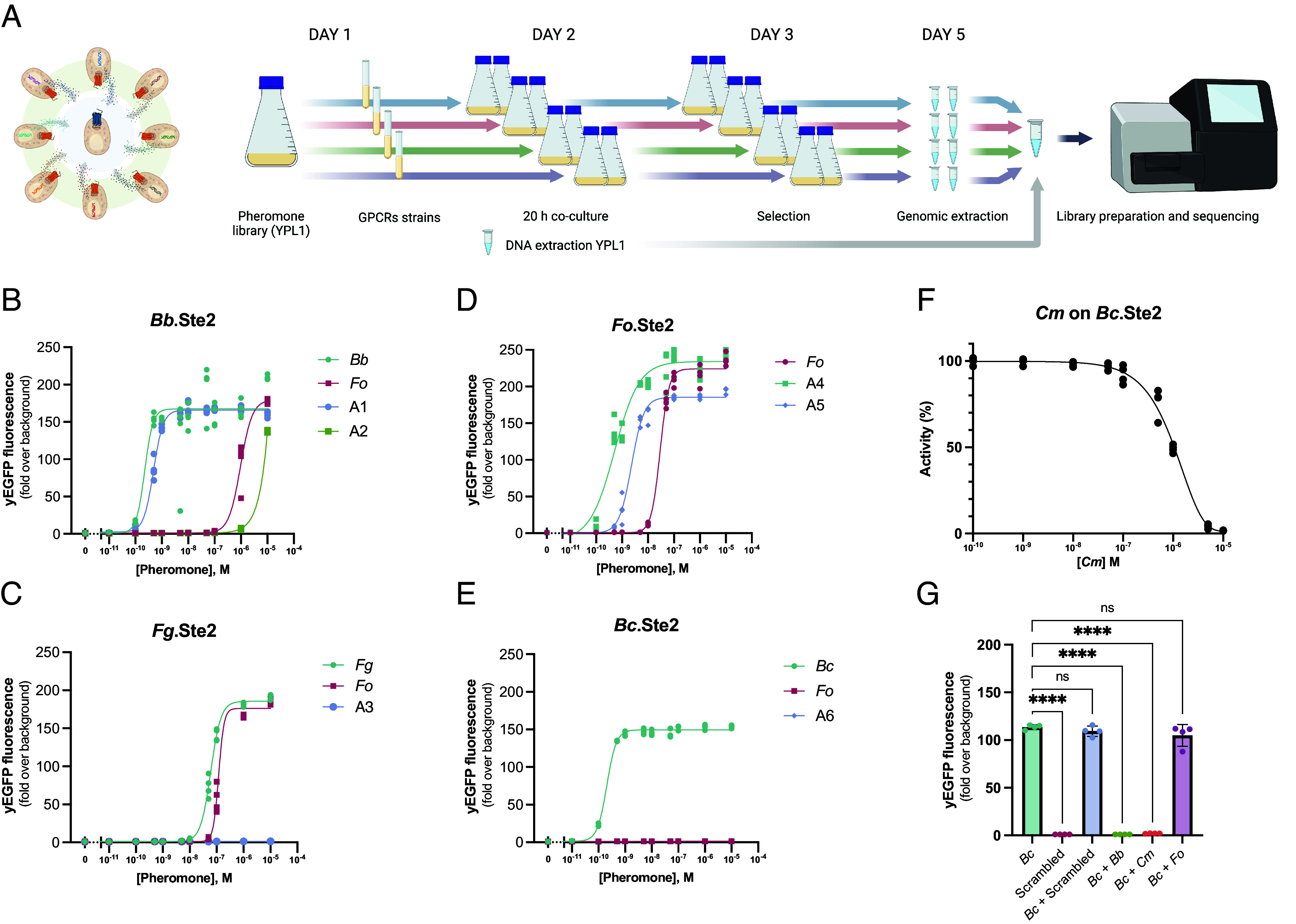
Identification of agonists and an antagonist by screening a library of pheromone variants. (*A*) A library of 8,000 alpha pheromone variants expressed in yeast (YPL1) was cocultured with yeast strains expressing GPCRs *Bb*.Ste2 (GEN88), *Fg*.Ste2 (GEN87), *Fo*.Ste2 (GEN90), or *Bc*.Ste2 (GEN89) for 20 h in shake flasks (2 flasks per GPCR). After 2 d of enrichment for diploid cells, gDNA was extracted and used for sequencing on NextSeq 500. (*B*–*E*) Dose–response curves of the most enriched pheromones in combination with (*B*) *Bb*.Ste2 (*Fo*, A1, and A2); (*C*) *Fg*.Ste2 (*Fo* and A3); (*D*) *Fo*.Ste2 (*Fo*, A4, and A5); and (*E*) *Bc*.Ste2 (*Fo* and A6) were compared with the cognate pheromone of each GPCR (*Bb*, *Fg*, *Fo*, and *Bc*, respectively). (*F*) Inhibition curve of *Bc*.Ste2 (GEN128) cocultured with 0.0005 μM of cognate *Bc* pheromone and increasing concentrations of *Cm* pheromone. (*G*) Fold change activation of *Bc*.Ste2 over nontreated control upon addition of 0.0005 μM *Bc* pheromone alone or in combination with 10 μM of either Scrambled (negative control), *Bb*, *Cm,* or *Fo* pheromone. 10 μM of Scrambled peptide alone was used as negative control. Note that *Bc* + Scrambled and *Bc* + *Fo* do not differ significantly from *Bc* alone, and that activation by *Bc* was completely abolished in the presence of *Bb* or *Cm* pheromones. Means represent four biological replicates. Statistical significance was determined using one-way ANOVA with Dunnett’s multiple comparison test in GraphPad Prism (****P < 0.0001).

To search for pheromone agonists within a mixed pool of variants, we cocultured the YPL1 library with cells individually expressing Ste2 GPCRs from the plant pathogens *Fg*, *Fo*, or *Bc*, or from the insect pathogen *Bb*. The culture was sequentially enriched for diploid cells before total DNA was extracted from the pool and analyzed by next-generation sequencing (NGS) (Fig. 2*A*). After normalizing the counts in reads per million (RPM) and subtracting the reads of the library before the cocultures, we observed a specific and exclusive enrichment for the *Bb* alpha pheromone in the *Bb*.Ste2 cocultures (+307 RPM) and for the *Fg* alpha pheromone in the *Fg*.Ste2 cocultures (+807 RPM). However, the *Fo* alpha pheromone was by far the most highly enriched sequence for the four Ste2 receptors tested in our screen (>340,000 RPM per sample).

To validate the YeMaP results, we performed dose–response curves with chemically synthesized versions of 10 different alpha pheromone sequences selected among the top 20 most abundant pheromone sequences obtained from the two biological replicates conducted for each GPCR (*SI Appendix*, Fig. S2 and Table S2). When incubated with the Ste2 GPCR for which they were originally enriched, seven out of the 10 selected peptides induced strong activation (≥150-fold increase over background) of the pheromone response pathway (Fig. 2 *B*–*E*), while two out of the remaining three pheromones induced low activation (≥1.5-fold increase over background) (*SI Appendix*, Fig. S3*A*).

The *Bb* and *Fo* alpha pheromones, which previously produced similar diploid frequencies (Fig. 1*D*), displayed two distinct half-maximal effective concentration (EC_50_) values in the *Bb*.Ste2 biosensor strain (Fig. 2*E*). The EC_50_ of the *Fo* pheromone was 3,910-fold higher than that of the Bb pheromone (*Fo*_EC_50_ = 9.58 × 10^−7^ M and *Bb*_EC_50_ = 2.45 × 10^−10^ M). We further noted that the alpha pheromone Agonist 1 (A1), which contains a conserved Arg4 residue preceded by Leu3 and followed by Pro5, a combination not found in any known organism (*SI Appendix*, Fig. S2), exhibited a potency similar to that of the *Bb* pheromone (A1_EC_50_ = 4.82 × 10^−10^ M). Furthermore, our screening identified two additional agonist pheromones, A4 and A5, both carrying a conserved Trp4, whose EC_50_ for *Fo*.Ste2 was at least 13-fold lower than that of the native *Fo* alpha pheromone (A4_EC_50_ = 5.16 × 10^−10^ M, A5_EC_50_ = 2.31 × 10^−9^ M, compared to *Fo*_EC_50_ = 2.92 × 10^−8^ M). While the A5 sequence was present in the *Fo* alpha factor prosequence (*SI Appendix*, Table S4), the A4 agonist contains an Ala5, representing a synthetic alpha pheromone with significantly increased potency, which requires 57 times less pheromone to achieve the same EC_50_ as the native *Fo* alpha pheromone (Fig. 2*D*).

Molecular dynamic (MD) simulations suggested that *Fo* pheromone, A5, and A4 share the same binding pose within the orthosteric pocket of *Fo*.Ste2, in which the carboxylic acid functional group of Trp10 forms salt bridges with Arg84 and Arg187 (*SI Appendix*, Fig. S4). We further noted that A4 had the most stable conformation from residues 5 to 10, especially in Trp10, which is the anchoring residue to the bottom of the orthosteric binding pocket (*SI Appendix*, Fig. S4*B*).

Next, we asked whether our YeMaP dataset could also detect potential alpha pheromone antagonists. By searching for underrepresented sequences in our analysis, we noted that a peptide encoded in the alpha factor prosequences of the entomopathogenic fungi *Cordyceps militaris* and *Beauveria asiatica* (*SI Appendix*, Table S4) was consistently lacking in the *Fg*.Ste2, *Fo*.Ste2, and *Bc*.Ste2 post mating enrichments, while its enrichment was observed for *Bb*.Ste2 (+7,508 RPM). Therefore, we speculated that this alpha pheromone (named *Cm*) could function as a potential GPCR antagonist that inhibits diploid formation. To test this idea, we obtained the chemically synthesized *Cm* pheromone and found that it triggered the activation of *Bb*.Ste2 and *Fo*.Ste2, but not of *Fg*.Ste2 or *Bc*.Ste2. When coincubated with the lowest pheromone concentration capable of inducing GPCR activation, *Cm* pheromone had an antagonistic effect on *Bc*.Ste2 (*SI Appendix*, Fig. S3*B*), with a half-maximal inhibitory concentration (IC_50_) of 1 μM (Fig. 2*F*). This IC_50_ was reached with a 2,000-fold excess of the antagonist compared to the agonist. A similar antagonistic effect was detected for the *Bb* pheromone during coincubation with *Bc*.Ste2, while no such effect was observed with the *Fo* pheromone (Fig. 2*G*). Interestingly, the two antagonistic pheromones *Cm* and *Bb* share eight out of nine residues with the *Bc* pheromone. Furthermore, the antagonistic effect was only observed when the two residues Arg4 and Pro5 (not present in *Fo* pheromone) were preceded by either Leu3 or Met3 instead of Gly3, combined with the presence of an additional Trp10 residue, which is lacking in the nine-residue *Bc* pheromone (*SI Appendix*, Tables S1 and S2). The antagonistic effect of the *Cm* pheromone was further confirmed in a mating assay. When a coculture consisting of strains expressing *Bc* GPCR and *Bc* pheromone was incubated with 50 μM of *Cm,* a two-fold reduction in diploid formation (*P* = 0.0071) was detected compared to the same coculture incubated with 50 μM of scrambled peptide (*SI Appendix*, Fig. S3*D*).

In conclusion, by using a straightforward “Library-on-One” YeMaP assay we identified alpha pheromone agonists as well as an antagonist, by choosing the most or least enriched sequences, respectively, followed by simple validation assays.

### Abiotic and External Factors Shape the Interaction Networks between Multiple Fungal Pheromone–GPCR Signaling Components (Library-on-Library).

**Fig. 3.**
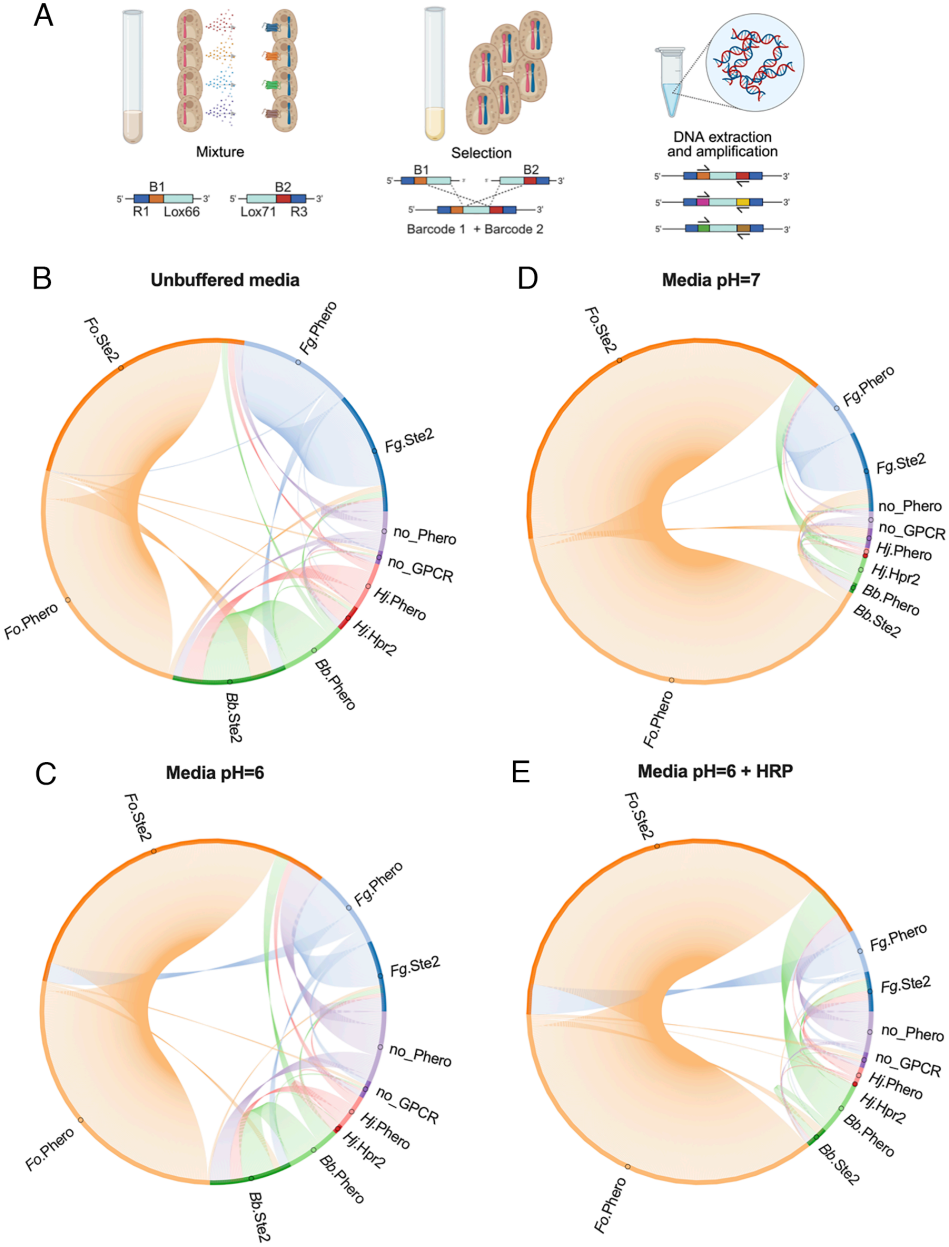
Abiotic and external factors govern fungal cell–cell communication mediated by GPCR–pheromone interactions. (*A*) Schematic illustration of the barcode system. *MAT***a** and *MAT*α cells contain a barcode flanked by a conserved region and a Cre-Lox recombination site on chromosome X. After successful mating the two barcodes can recombine and become located on the same chromosome. B-E Chord diagrams of consortia combining yeast strains expressing either *F. graminearum* (*Fg*), *F. oxysporum* (*Fo)*, *H. jecorina* (*Hj*), *B. bassiana* (*Bb*) Ste2 GPCRs with strains individually secreting the respective cognate alpha pheromones, as well as the two negative controls (empty strains) in the following media: (*B*) Unbuffered pH = 5.6; (*C*) buffered at pH = 6; (*D*) buffered at pH = 7; and (*E*) buffered at pH = 6 supplemented with 1 μM Horse Radish peroxidase (HRP). Dark color represents the GPCR-expressing strain while the lighter version represents the strain secreting the cognate alpha pheromone. Orange = *Fo*, Blue = *Fg*, Green = *Bb*, Red = *Hj*, Violet = negative control with empty constructs. Arcs represent the connection between a GPCR and a pheromone-expressing strain. The size of an arc reflects the average relative abundance of a diploid cell. Relative quantification was done by comparing the cycle threshold (Ct) value of every possible combination of two barcodes with the Ct value of the conserved region flanking all barcodes. All experiments were conducted in three biological replicates.

External parameters such as pH or nitrogen source act as potent regulators of fungal growth, development, and pathogenicity (33). Here, we analyzed the effect of such abiotic factors on fungal GPCR-based cell–cell communication in multiplex (Fig. 3*A*). To this aim, we introduced a barcode system based on the Cre-lox recombinase (34), which allows to genetically detect the occurrence of mating between two yeast strains, each expressing a given Ste2 GPCR or an alpha pheromone. In brief, each haploid cell contains a conserved region flanked by a unique barcode of 20 bp, a spacer, and a Lox site (*SI Appendix*, Table S5). After successful mating, a unidirectional recombination event positions the two barcodes on the same chromosome (35) (Fig. 3*A*). qPCR was used to detect the presence of the two barcodes and reveal the association between any given pheromone and GPCR, allowing us to estimate the relative abundances of diploid cell types in a pool. The relative abundance of a diploid combination was compared with the abundance of all diploids containing the conserved regions (R1 + R3) flanking the two barcodes (*Materials and Methods*).

We first tested the specificity of the system by running four different cocultures and combinations of these cocultures in consortia, by assessing whether the two barcode sites were on the same chromosome (*SI Appendix*, Fig. S5). The strains lacking constructs (no_Pheromone, no_GPCR) were capable of forming diploids in coculture (Fig. 1*D* and *SI Appendix*, Fig. S5), but were not detected in consortia with other strains (*SI Appendix*, Fig. S5). Introduction of barcodes in the remaining strains expressing pheromones or GPCRs allowed specific amplification and quantification of diploids by qPCR. From all cocultures tested, the relative abundance of diploids detected with each set of primers was comparable (*SI Appendix*, Fig. S6 and Dataset S3).

To mimic the presence of multiple fungal organisms of ecological interest, we performed consortia combining yeast strains expressing either *Fg*, *Fo*, *Hj*, *Bb* Ste2 GPCRs with strains individually secreting the respective cognate alpha pheromones, as well as the two negative controls (empty strains). These 10 different strains were combined into preculture tubes under different conditions, including three different pHs (unbuffered, pH = 6, or pH = 7), or in the presence of HRP (pH = 6 + HRP) to mimic a plant root environment (11). Interestingly, the *Fo* pheromone–GPCR system was the most robust and consequently more effective in the formation of diploid cells across all the conditions tested (Fig. 3 *B*–*E*). Conversely, in the *Fg* pheromone–GPCR system which had the second most abundant diploid formation in unbuffered media or in media buffered at pH = 6 or pH = 7, the presence of HRP significantly reduced the formation of diploid cells (*P* = 0.023, *SI Appendix*, Fig. S7*A*).

Overall, with the exception of *Fo* GPCR and *Fo* pheromone-expressing strains, a reduction in diploid formation was observed at increasing pH values or in the presence of HRP (*SI Appendix*, Fig. S7*A*). Importantly, the results of this library-on-library approach were consistent with those from individual cocultures (one-on-one) containing only one strain expressing a given GPCR and another secreting a given alpha pheromone from *Fg*, *Fo*, *Bb*, or *Hj* (*SI Appendix*, Fig. S7*B*).

In contrast to the above approach, the yeast biosensor assay failed to detect a difference in signaling behavior between *Fo*.Ste2 and the remaining GPCRs when exposed to HRP. We observed activation of the control strain lacking a GPCR in unbuffered Synthetic Complete (SC) media supplemented with 2 μM HRP, as well as a general activation (≈1.5-fold increase in yEGFP fluorescence) at pH = 6 with 2 μM HRP, both in the control strain and in all the biosensor strains tested (*SI Appendix*, Fig. S8 *A* and *B*).

Taken together, these results demonstrate that a synthetic mating platform can be used to simultaneously study the effect of abiotic factors on cell–cell communication between different fungal GPCRs and alpha pheromones. Furthermore, we found that the library-on-library approach leads to similar results as the one-on-one approach, and that *Fo* GPCR–pheromone communication was not affected by the different external factors tested.

### External Pheromone Supplementation Alters the Cell–Cell Communication of Phytopathogen GPCRs in a Fungal Consortium.

After using the YeMaP to determine the effect of abiotic factors on four different fungal GPCR–pheromone pairs and showing the robustness of the *Fo* signaling components, we next explored whether external pheromone addition can reshape a network of pheromone–GPCR interactions. With the goal of inhibiting the pheromone–GPCR systems from the two plant pathogens *Fo* and *Fg* (36) and promoting those from the non-pathogenic/commensal fungi *Bb* and *Hj* (37, 38) we tested the effect of exogenously supplementing either *Cm*, A4, or scrambled peptide.

**Fig. 4.**
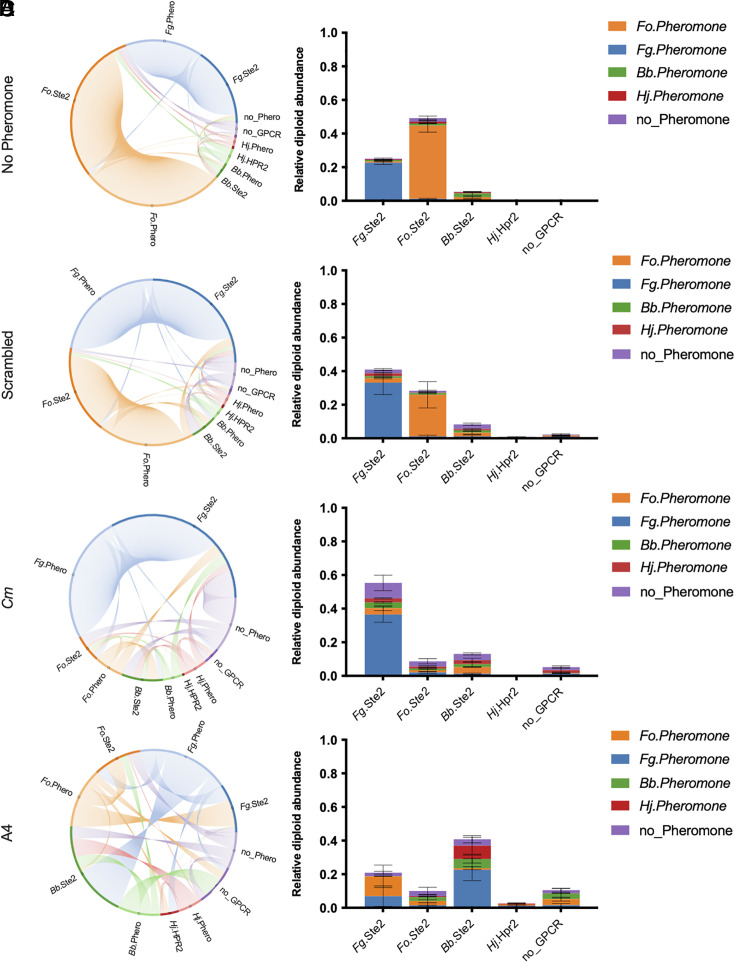
GPCR-mediated cell–cell communication in a consortium can be controlled by external pheromone supplementation. Starting from a consortium in SC media without external supplementation of pheromone in (*A*), we added 10 μM of either scrambled peptide (negative control) (*B*), *Cm* pheromone (*C*), or A4 pheromone (*D*). On the left, in the chord diagrams a dark color represents the GPCR-expressing strain while the lighter version represents the strain secreting the cognate pheromone. Orange = *Fo*, Blue = *Fg*, Green = *Bb*, Red = *Hj*, Violet = negative control with empty constructs. Every arc represents the connection between a GPCR and a pheromone strain. The size of an arc reflects the average relative abundance of a diploid cell. Relative quantification was done by comparing the Ct value of every possible combination of two barcodes with the Ct value of the conserved region flanking all barcodes. On the right, the bar plots represent the distribution of pheromone secreting strains that have successfully mated with each of the five GPCR-expressing strains. All experiments were conducted in three biological replicates.

In SC media without external pheromone supplementation or containing 10 μM of scrambled peptide, almost all diploids detected were formed from the *Fo*.Ste2 + *Fo*.Pheromone or *Fg*.Ste2 + *Fg*.Pheromone pairs (Fig. 4 *A* and *B*). By contrast, external addition of *Cm* pheromone resulted in a 0.2-fold decline in diploids containing *Fo*.Ste2 (*P* = 0.001) concomitant with a 2.2-fold increase in diploids containing *Fg*.Ste2 (*P* = 0.015), while addition of A4 peptide triggered an 8.7-fold or 12.1-fold increase in formation of diploids containing *Bb*.Ste2 (*P* = 0.004) or *Hj*.Hpr2, respectively (*P* = 0.001), together with a 19-fold increase in the negative control lacking a GPCR (*P* = 0.013) (Fig. 4 *C* and *D*).

A4 peptide supplementation reduced the presence of diploids with *Fo*.Ste2 by 0.2-fold (*P* = 0.001), without increasing the presence of diploids containing *Fg*.Ste2. *Fg*.Ste2 and *Fg*.Pheromone interaction was reduced by 0.3-fold, however, *Fo.*Pheromone strain compensated for the final amount of diploids containing *Fg*.Ste2 (*SI Appendix*, Fig. S9*A*).

Furthermore, media with different nitrogen sources, which are commonly used as fertilizers, affected the relative distribution of diploid abundances. Compared to SC media with ammonium sulfate alone (SC), SC media supplemented both with ammonium sulfate and Urea (SC-AS/Urea) led to a fourfold and 20-fold increase of diploids containing *Bb*.Ste2 and *Hj*.Hpr2, respectively (*SI Appendix*, Fig. S9*B*). By contrast, the presence of urea did not affect the abundance of diploids with *Fo*.Ste2 or the control strain with no_GPCR, but led to a 0.8-fold decrease in the abundance of diploids with *Fg*.Ste2 (*SI Appendix*, Fig. S9*B*).

In summary, YeMaP reconstructed a network of interactions between different fungal alpha pheromones and GPCRs, enabling us to test how external pheromone supplementation could interfere with GPCR-mediated cell–cell communication in fungal pathogens.

### YeMaP Findings Are Reproduced In Vivo in the Phytopathogen *F. oxysporum*.

**Fig. 5.**
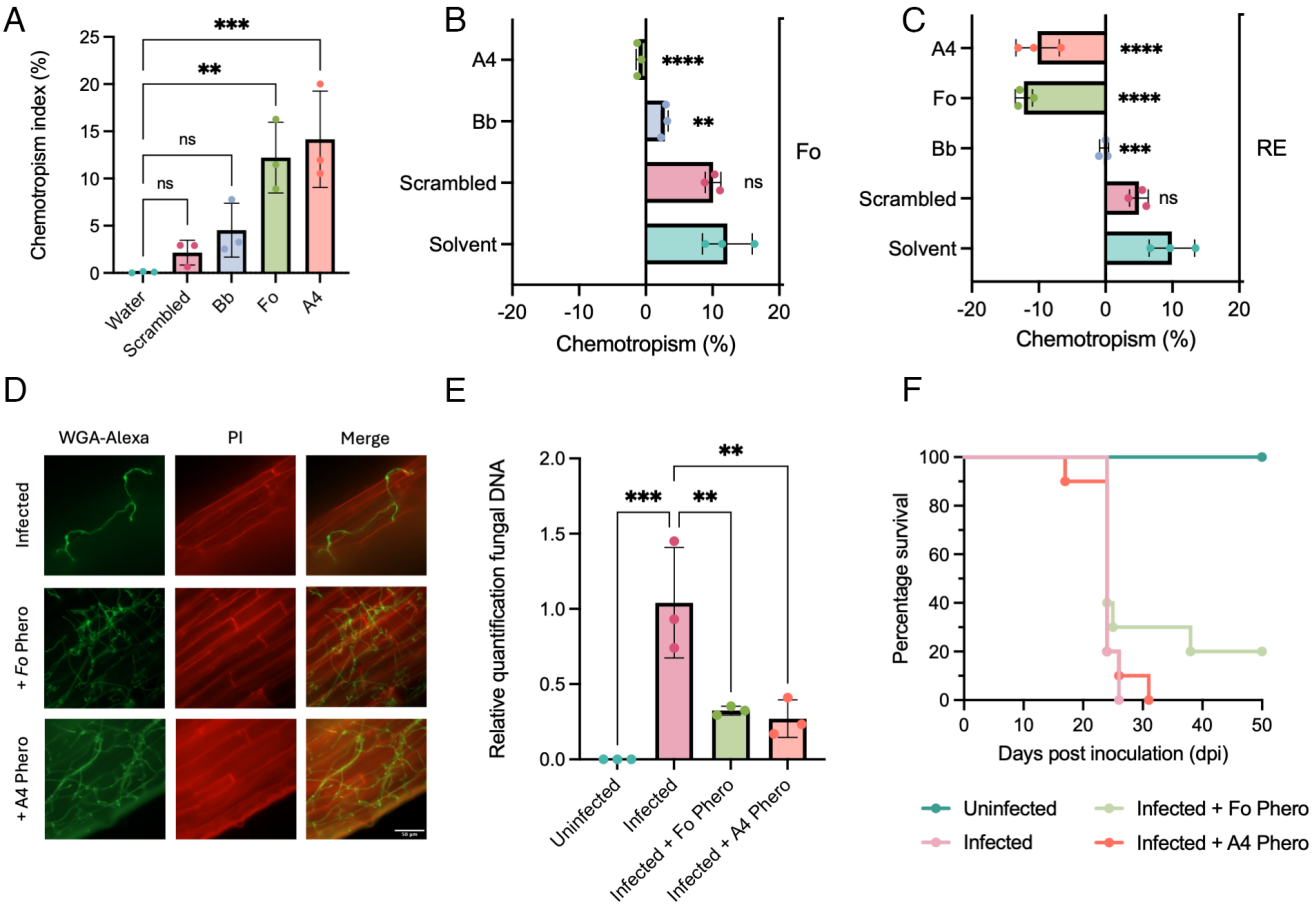
YeMaP-selected pheromones trigger *F. oxysporum* hyphal chemotropism and interfere with plant root penetration. Directed growth of *F. oxysporum* germ tubes embedded in water-agar after 13 h exposure to gradients of the indicated compounds. (*A*) Directed growth toward gradients of 400 μM of the following pheromones: scrambled peptide (neg. control), *Bb* (*B. bassiana*), *Fo* (*F. oxysporum*), or A4 (Agonist 4). All pheromones were tested against the solvent (50% methanol). (*B*) Simultaneous exposure to competing gradients of 400 μM *Fo* pheromone, opposed to either solvent or 400 μM scrambled peptide, *Bb* pheromone, or A4. (*C*) Simultaneous exposure to competing gradients of tomato RE, opposed to either solvent or 400 μM scrambled peptide, *Bb* or *Fo* pheromone, or A4 peptide. (*D*) Fluorescence microscopy of tomato roots subjected to the indicated treatments, 3 d after inoculation with microconidia of *F. oxysporum*. Fungal cell walls were stained with WGA-Alexa Fluor 488 (green) while plant cell walls were stained with propidium iodide (PI) (red). (Scale bar, 50 μm.) (*E*) Fungal DNA in inoculated tomato roots was measured by qPCR of the *Fol4287*-specific *actin* gene using total DNA extracted 3 d after inoculation. Fungal burden was calculated using the 2^−ΔΔCt^ method and normalized to the tomato *gapdh* gene. (*F*) Kaplan–Meier plot showing survival of groups of 10 tomato plants (cv. Momotaro) inoculated by pipetting on top of the roots a suspension of 3.2 × 10^6^ microconidia/mL supplemented either with the solvent (infected), or with the indicated pheromone (infected + Fo phero, infected + A4 phero). Water without microconidia was used as negative control (uninfected). Data shown are from one representative experiment. The experiment was performed twice with similar results. In *A*–*C* and *E*, means represent three biological replicates. Statistical significance was determined using one-way ANOVA with Dunnett’s multiple comparison test in GraphPad Prism (**P ≤ 0.01, ***P ≤ 0.001, ****P ≤ 0.0001).

To validate our YeMaP findings in an in vivo fungal system, we tested how external pheromone supplementation affects GPCR-dependent functions in the plant pathogen *F. oxysporum.* As previously reported (10) we found that exogenous addition of 400 μM alpha pheromone from *Fo* or *Bb*, or of the synthetic agonist A4, caused a significant reduction of *F. oxysporum* microconidia germination (*SI Appendix*, Fig. S10). We next tested the chemoattractant activity of the different pheromones using an established chemotropism assay (11). Interestingly, *Fo* and A4, the two peptides selected in the mating enrichment screen with the YeMaP (*Library-on-One*), induced a significant chemotropic response in *F. oxysporum* germ tubes, whereas the *Bb* pheromone and the scrambled peptide did not (Fig. 5*A*). Furthermore, when *F. oxysporum* microconidia were exposed to two competing chemoattractant gradients, A4 fully abolished hyphal chemotropism toward *Fo* pheromone, while *Bb* pheromone only achieved a partial reduction (Fig. 5*B*). Both A4 and *Fo* pheromone were able to invert directional growth of *F. oxysporum* germ tubes toward tomato root exudate (RE), while *Bb* pheromone annulled the RE response and scrambled peptide had no significant effect (Fig. 5*C*).

Given their strong chemoattractant effect, we hypothesized that *Fo* and A4 pheromone could interfere with *F. oxysporum* infection in tomato roots. While exogenous addition of *Fo* or A4 pheromone led to increased adhesion of the *F. oxysporum* germ tubes, either among themselves or to tomato roots (*SI Appendix*, Fig. S12 and Fig. 5*D*), after careful washing of the root surface (*SI Appendix*, Fig. S11 *A* and *B*) a significant reduction in fungal biomass was detected in inoculated roots treated with *Fo* or A4 pheromone compared to the untreated infected roots (Fig. 5*E*). Despite this short-term reduction in fungal biomass within the root, no significant difference in plant survival between the treatments was observed after 50 d (Fig. 5*F*).

In conclusion, the in vivo experiments confirmed the utility of the YeMaP in the identification of bioactive pheromones, and in particular, an artificial agonist with improved properties on chemoattraction of *F. oxysporum* germ tubes and in reduction of fungal root penetration during early stages of infection.

## Discussion

Fungal hyphae can sense a variety of chemical cues, allowing them to grow toward nutrient sources, mating partners, or host organisms (39). Here, we established YeMaP, a synthetic YeMaP able to decipher fungal GPCR–pheromone interactions. We used the platform to investigate how such interactions are affected by external factors or can be manipulated through exogenous pheromone supplementation.

YeMaP exhibited an increased resolution compared to the standard designs for yeast biosensors (13, 40, 41) (Fig. 1 *E* and *F*). This improvement can be attributed to the well-documented role of the Sst2 protein in suppressing signaling noise and GPCR activation (42–44). Accordingly, GPCR signaling did not always translate to a comparable mating efficiency. For example, from the pheromone library screen (*Library-on-One*), one of the most enriched pheromones (A4) and one of the least enriched pheromones (*Cm*) for *Fo*.Ste2, resulted in being agonists for *Fo*.Ste2 (Fig. 2*D* and *SI Appendix*, Fig. S3*C*) and consequently affected *Fo*.Ste2 activity in Library-on-Library interaction networks (Fig. 4 *C* and *D*).

We used YeMaP to successfully screen a library of 8,000 pheromone variants in a single test tube, and identified the *Fo* pheromone as the most enriched for the four different Ste2 GPCRs tested. Even though we observed enrichment for the cognate pheromones, the presence of a mixture of different pheromones and synthetic peptide sequences could have led to an advantage for those with higher EC_50_ values (*Fo*, A1, and A2 in *Bb*.Ste2, *Fo* in *Fg*.Ste2) or almost no effect such as A3 in *Fg*.Ste2 (Fig. 2 and *SI Appendix*, Fig. S3*A*). For *B. cinerea* Ste2, a GPCR that evolved to sense a pheromone with 9 residues instead of 10, we detected the antagonistic effect of two alpha pheromones from two different fungi, *C. militaris* and *B. bassiana* (Fig. 2 *F* and *G*). However, to reach an IC_50_ the antagonist pheromones need to be added in 2,000-fold excess compared to the agonist (Fig. 2*F*), a situation that may not be present in natural settings. The necessity for such high concentrations of antagonist pheromone could be partially explained by the type of assay used to test the antagonist effect on a yeast biosensor. The standard yeast biosensor strain (40) has high expression levels of the fungal GPCR and Gα subunit, besides carrying several gene knockouts (in particular *sst2Δ*) to create a highly sensitive system suitable to detect low concentrations of agonist. Such a setting may not be ideal to screen for potential pheromone antagonists. Second, the antagonistic effect of *Cm* pheromone was maintained in the mating assay (*SI Appendix*, Fig. S3*D*), but only at high concentrations (50 μM), suggesting that it is a weak antagonist of *Bc*.Ste2. Future studies are required to improve the selection of pheromone antagonists and validate their effect in different fungal systems.

YeMaP successfully captured the complexity of multiple GPCR–pheromone interactions and detected the effect of different environmental factors such as pH, nitrogen sources, or plant peroxidase on cell–cell communication (*Library-on-Library*). This allowed us to confirm the superior robustness of the *Fo*.Ste2-alpha pheromone interaction compared to those from *Fg*, *Bb*, or *Hj* (Fig. 3). This finding may be of biological relevance, since *F. oxysporum* was shown to secrete alkalinizing peptides during plant infection, thereby actively reshaping the extracellular pH and enhancing plant root colonization (45). At the same time, plant peroxidases trigger germ tube chemotropism via *Fo*.Ste2 (11), a GPCR which also controls microconidia germination (10) and fusaric acid production (46). We speculate that *F. oxysporum* may have evolved a GPCR–pheromone system that remains functional across the different environments encountered during plant infection (Fig. 3 and *SI Appendix*, Figs. S7 *A* and *B* and S9*B*). Although both *F. oxysporum* and *F. graminearum* can sense plant roots (11) or plant exudates (47) through their mating receptors, our results indicate that the underlying mechanisms may be functionally distinct. In the presence of HRP, yeast strains expressing *Fo* components were able to maintain the GPCR–pheromone communication, while those expressing *Fg* components were not (*SI Appendix*, Fig. S7 *A* and *B*).

Importantly, our YeMaP results with the *Fo*.GPCR correlated with real hyphal chemotropism in *F. oxysporum*. Two of the best hits selected from the YeMaP pheromone enrichment experiment against *Fo*.Ste2, *Fo* alpha pheromone and the synthetic agonist peptide A4, exhibited a strong chemoattractant effect toward germ tubes of *F. oxysporum* (Fig. 5 *A*–*C*). In contrast to YeMaP, the yeast biosensor assay was unable to discriminate between pheromones capable of inducing a strong (*Fo* and A4) or a weak chemotropic response (*Bb*) in *F. oxysporum* (Figs. 1*E* and 5 *A*–*C*). At the same time, in the one-on-one YeMaP experiment, we observed promiscuity of the *Bb*.Ste2 GPCR, particularly toward the *Fo* pheromone (Fig. 1 *C* and *E*). Our results also indicate that *Fo* pheromone can induce germ tube chemotropism in *B. bassiana* (*SI Appendix*, Fig. S1). Previous studies suggested a role of *B. bassiana* in controlling *F. oxysporum* infection, increasing tomato plant survival rate (48, 49). Further investigations are needed to determine whether pheromone–GPCR signaling could contribute to the biocontrol activity of *B. bassiana* against *F. oxysporum*.

We used YeMaP to test whether external addition of pheromone interferes with signaling through a GPCR–pheromone pair of interest. Our results are in accordance with previous studies highlighting the importance of the pheromone gradient for successful mating in yeast (25, 50, 51). Furthermore, YeMaP allowed to test in a single tube the specific effect of a supplemented pheromone against four different GPCRs which recognize pheromone peptides with a similar backbone (Fig. 4). External pheromone supplementation in the YeMaP led to a significant reduction of diploids containing *Fo*.Ste2, indicating that it interfered with communication between a yeast cell secreting *Fo* alpha pheromone and its mating partner expressing the GPCR (Fig. 4 *C* and *D*). Similarly, supplementation of Fo pheromone or A4 peptide to *F. oxysporum* microconidia enhanced hyphal adhesion (Fig. 5*D*), but reduced the ability of the pathogen to penetrate tomato roots (Fig. 5*E*). These results indicate that alpha pheromone could be secreted by *F. oxysporum* during early stage of germination (10) and subsequently be degraded actively after plant recognition. In support of this hypothesis, analysis of previously published transcriptomics data from early infection stages of *F. oxysporum* (52) detected upregulation of genes functioning in the pheromone response and the cell wall integrity (CWI) MAPK cascades (*SI Appendix*, Fig. S13). Additionally, the aspartyl protease Bar1, which specifically degrades *F. oxysporum* alpha pheromone (10), was significantly upregulated (|Log_2_ fold change| ≥ 2.5, *P* ≤ 0.0001), while the pheromone genes did not show significant changes in transcript levels during plant infection (*SI Appendix*, Fig. S13). While external pheromone addition significantly reduced fungal entry into tomato roots, we did not observe a clear effect on plant mortality (Fig. 5*F*). This could be due to the fact that external pheromone was only supplemented once during initial root inoculation, and its stability and consequently action was insufficient to provide protection during the entire duration of the infection assay (50 d).

In the future, we envision using YeMaP as a model for studying how different soil compositions and fertilizers influence cell–cell communication within fungal consortia and for developing designed molecules to control fungal pathogen virulence by perturbing their natural ability to engage in cell–cell communication.

## Materials and Methods

All materials, experimental data, and statistics can be found in Datasets S1–S3.

### Molecular Cloning for Yeast Engineering.

#### Gene blocks and DNA parts.

Gene blocks (Integrated DNA Technologies - IDT) gDOG1 to gDOG10 (Dataset S1) were codon optimized for *S. cerevisiae* with the IDT Codon Optimization tool. All oligos and gene blocks were purchased from IDT.

#### PCR and DNA handling.

Plasmids were assembled with USER cloning (53) to be compatible with the EasyClone-MarkerFree system for CRISPR/Cas9 engineering (54) and adapted to the MAD-cloning strategy (55). All plasmids, oligos, gBlocks, and parts are listed in Dataset S1.

#### Bacterial handling for plasmid construction and propagation.

Plasmids were transformed (heat-shocked) in *Escherichia coli* DH5α strain. The competent cells were stored in glycerol at −70 °C and cultivated in Terrific Broth with ampicillin (100 mg/L) at 37 °C in liquid media at 300 rpm or on agar plates for 16 to 20 h.

### Strains, Media, and Transformation.

#### Strains.

Strain CEN.PK2-1C and BY4741 (EUROSCARF) were used as yeast background strains. All yeast strains can be found in Dataset S1. *B. bassiana* (DSM 62075) strain was obtained from the Leibniz Institute DSMZ-German Collection. All the experiments with *F. oxysporum* were conducted using *F. oxysporum* f. sp. *lycopersici* 4287 (*Fol4287*).

#### Media.

Yeast strains were grown in SC media with 2% (w/v) glucose with a pH = 5.6 or in buffered media with ammonium sulfate, urea, and the citrate phosphate buffer system (0.1×) following Prins et al. protocol (56).

For *F. oxysporum* microconidia production, the strain was grown for 3 to 4 d in liquid potato dextrose broth (PDB) at 28 °C and 170 rpm. Germination media (10) was used for assessing the spore germination. RE was prepared by removing 2-wk-old tomato plants from vermiculite and washing the roots to remove any adhering substrates (11).

#### Transformation.

Except for the Yeast pheromone library (YPL1; see below), all strains were generated using the LiAc/ssDNA/PEG method (57). All genetic cassettes were integrated into the genome of strains harboring pEDJ391 to express CRISPR-Cas9 (58). Genomic integration was achieved with the cotransformation of a *Not*I linearized fragment with homology arms and a gRNA helper vector targeting the desired EasyClone site (54). About 1 to 2 μg for each plasmid or fragment of DNA was used in all chemical transformations of *S. cerevisiae*. The strain was selected in SC with 2% (w/v) glucose media lacking the appropriate amino acid or with the addition of 100 mg/L nourseothricin (Jena Bioscience, Cat.#AB-101).

#### Yeast pheromone library (YPL1).

YPL1 was generated by PCR amplifying two fragments F1 (of 1,978 bp generated with XI-3 UP FW + DOG105) and F2 (of 961 bp generated with DOG106 + XI-3 RV) with 129 bp of homology between each other. The plasmid pDOG44 was used as a template (*SI Appendix*, Table S3). The strain GEN74 was streaked on a SC-leu plate and treated for electroporation following the Wäneskog et al. protocol (32). 400 μL of the yeast solution was used per transformation reaction by mixing the cell suspension with 5 µg of F1, 5 µg of F2, 5 µg of pESC_URA_gRNA_XI-3 plasmid and 10 μL (100 µg) of boiled single-stranded salmon sperm carrier DNA (ssDNA) in an Eppendorf tube. The library was expanded in SC -leu -ura for 48 h and aliquots were stored in glycerol stock at −70 °C. We avoid the introduction of a second pheromone sequence in the diploid cells by introducing a mutation in the XI-3 site of the GPCR strains.

## Experimental Procedures

### Mating Trials.

#### One-on-One in a tube.

*MAT***a** and *MAT*α strains were inoculated from a cryostock into a culture tube with 1 mL of SC -leu and SC -his respectively (30 °C, 250 RPM, overnight). Reaching a final OD = 0.1 with a 1:1 ratio around 5 μL from a saturated *MAT***a** culture and 5 μL from a saturated *MAT*α culture were combined into a culture tube with 1 mL of SC media and for 20 h (30 °C, 250 RPM). The haploids were plated in SC -leu (*MAT***a** strains) and SC -his (*MAT*α strains) in 3 plates per strain. After 20 h the diploid cells were appropriately diluted and plated in SC -leu -his plates. The colonies on plates were enumerated after 4 d of incubation at 30 °C using the Count This app installed on an iPhone.

#### One-on-One in a deep well plate.

Similarly to the One-on-One in tube assay 2 μL from a saturated *MAT***a** culture and 2 μL from a saturated *MAT*α culture were combined into 250 mL of SC media and incubated at 30 °C in a shaking incubator for 20 h (30 °C, 250 RPM). Enumeration of haploid and diploid cells was conducted in the same way.

#### Library-on-One: GPCR against the yeast pheromone library.

Two full cryotubes of the yeast pheromone library (YPL1) were inoculated into a flask containing 50 mL of SC -leu. GEN87 (*Fg*.Ste2), GEN88 (*Bb*.Ste2), GEN89 (*Bc*.Ste2), and GEN90 (*Fo*.Ste2) were inoculated from the cryostock in four different tubes containing 4 mL of SC -his (30 °C, 250 RPM). YPL1 was cocultured with each single receptor in a shake flask with 50 mL of SC in two biological replicates with a final OD = 0.2 and a ratio of *MAT***a** 1:1 *MAT*α. After 20 h 1 mL of the coculture was washed twice with Milli-Q water and transferred into a flask with 50 mL of SC -his -leu (30 °C, 250 RPM). The culture was left growing for 48 h (30 °C, 250 RPM) and 5 mL of it was used to perform a genome extraction using the yeast DNA extraction kit (ThermoFisher).

#### Library-on-Library: Consortia of strains with fungal pheromones and GPCRs.

*MAT***a** and *MAT*α strains were individually inoculated from a cryostock into culture tubes with 1 mL of SC -leu and SC -his, respectively (30 °C, 250 RPM, overnight). All *MAT***a** and *MAT*α strains were mixed in two different tubes by adding the same amount of volume from the starting tubes. 50 μL of *MAT***a** cells and 50 μL of *MAT*α cells were combined in culture tube to reach a final volume of 5 mL (SC media) and incubated at 30 °C in a shaking incubator for 20 h (30 °C, 250 RPM). 50 μL of the SC culture were transferred to a culture tube containing SC-his-leu reaching a final volume of 5 mL. After 48 h, genomic DNA was extracted from the SC -his -leu using the Yeast DNA extraction kit (ThermoFisher).

#### qPCR protocol: Yeast library-on-library.

From the *Library-on-Library* experiments, 50 ng of genomic DNA was used as a template to detect the relative abundance of diploid formation. Each condition was tested in three biological replicates. All qPCRs were performed using SYBR Master Mix (ThermoFisher) on the QuantStudio 5 system.

#### Handling of synthetic pheromones.

Synthetic peptides (*SI Appendix*, Table S2) were purchased by GenScript Biotech (4 mg, ≥95% purity) and dissolved in 100% DMSO to a concentration of 1,000 μM, then 10-fold diluted in SC media to 1,000 µM (10% DMSO). A serial dilution in SC + 10% DMSO was made to achieve a concentration of 1 × 10^−11^, 1 × 10^−10^, 5 × 10^−10^, 1 × 10^−9^, 5 × 10^−9^, 1 × 10^−8^, 5 × 10^−8^, 1 × 10^−7^, 1 × 10^−6^, 1 × 10^−5^ M for the dose–response curve.

For the *B. bassiana* and *F. oxysporum* chemotropism assay and plant infection assays the pheromones were dissolved in 50% (v/v) MeOH to reach a concentration of 4,000 μM.

#### HRP preparation.

HRP (Sigma P-8375) was resuspended in phosphate-buffered saline buffer (PBS) at a concentration of 200 µM.

#### Dose–response curves and flow cytometry.

Characterization of the fungal pheromones was done by conducting dose–response analyses following Jensen et al. protocol (20). The same procedure was followed for studying the antagonist effect of a pheromone with one exception. The biosensor strains were first incubated with the candidate antagonist pheromone (priming), followed by the addition of the agonist pheromone after 15 min (competition). For each condition tested, four biological replicates were analyzed, with a threshold of 10,000 events per replicate.

#### Quantification of fungal chemotropism.

*Fungal* chemotropism was conducted as previously described (11). For each compound or combination of compounds tested, 300 hyphal tips were scored. All experiments were performed in three biological replicates.

#### Tomato plant growth conditions.

Tomato seeds (*Solanum lycopersicum* cv. Moneymaker from EELM-CSIC; susceptible to *F. oxysporum f. sp. lycopersici* race 2) were surface sterilized by immersion in 20% bleach (v/v) for 30 min and sown in moist vermiculite. The seeds and plants were maintained in a growth chamber under the following conditions: 28 °C, 40 to 70% relative humidity and a photoperiod of 14 h of 36 W white light and 10 h of darkness.

#### Tomato root inoculation and fluorescence microscopy of infected roots.

For microscopic observation of *F*. *oxysporum* during tomato root infection, roots of 2-wk-old tomato seedlings were inoculated with *F*. *oxysporum* microconidia. Freshly obtained microconidia were resuspended in sterile water at a concentration of 3.2 × 10^6^ microconidia/mL were mixed in Eppendorf tubes and mixed either with the pheromone of interest at the desired concentration or with the solvent treatment in a volume ratio 9 microconidia:1 pheromone volume. For each condition tested, three tomato plants were placed on a water agar plate, and 5 μL of the microconidia suspension were applied on 10 different spots on each tomato root (in total 50 μL per root). After 30 min at room temperature the roots were dried on a paper towel, transferred to a new water agar plate and incubated 3 d at 28 °C. Then the roots of three plants were pooled together and stained (*SI Appendix*, Fig. S11*A*) with 20 µg/mL PI (Sigma-Aldrich) and 10 µg/mL WGA-Alexa Fluor 488 (Invitrogen) as previously described (59). Wide-field fluorescence imaging was performed with a Zeiss Axio Imager M2 microscope equipped with a Photometrics Evolve EMCCD camera, using the 40× oil objective.

#### Quantification of fungal DNA in tomato roots.

Tomato roots inoculated with *F*. *oxysporum* microconidia and incubated 3 d as described above. Roots were washed three times using a water washing bottle with Driplok vapor venting (*SI Appendix*, Fig. S11*B*). Total DNA was extracted from tomato roots, and *F. oxysporum* biomass was measured by qPCR as previously described (60) using primers specific for the *F. oxysporum actin* gene. For each biological replicate, roots from three plants were pooled for DNA extraction. Relative fungal biomass was calculated using the 2^−ΔΔCt^ method, with primers for the *Fol4287 actin* gene (FOXG_01569) normalized to the tomato *gadph* gene.

#### Tomato plant mortality assay.

Tomato plant infection assays were performed as described (61) with *F*. *oxysporum* microconidia and treatment inoculation as described above. Plants were transferred to a growth and plant survival was recorded daily for 50 d. Plant death was diagnosed as a complete collapse of the stem, without any green parts left. The Kaplan–Meier test was used to assess statistical significance of differences in survival among groups using the log-rank test in GraphPad Prism. The infection experiment was conducted two times with 10 plants per condition.

### Illumina.

#### Illumina yeast library preparation pre- and postenrichments.

Following Illumina’s recommendation protocol, 50 ng of high-quality gDNA was used for a two-step PCR. PCR clean-up was performed with AMPure XP beads (1.8 ratio) and EtOH 80%. Bioanalyzer DNA 1000 chip was used to verify the correct size of the amplicons. The following primers were used for barcoding: Nextera XT Index Primer 1 (i701, i702, i703, i704, i705, i706, i707, i708, or i709), with Nextera XT Index Primer 2 (i501). Final DNA concentration was measured using Qubit 2.0. A pool of all samples was conducted to achieve a final DNA concentration of 10 nM with a proportion of 55 % YLP1 pre-enrichment and the remaining 45% with the equimolar concentration of all the enriched samples.

#### Illumina dAta processing yeast library preparation pre- and postenrichments.

The sample was sequenced with NextSeq 500 Mid Output 150 cycles with four technical replicates. Amplicon analysis was conducted with the standardized workflow Natrix (62). We combined the raw reads of the technical replicates and performed reads per million (RPM) normalization. Furthermore, we subtracted the RPM counts of the control starting library to allow for a better sample comparison. The data processing and ASV count analysis were done in Jupyter notebooks which can be found at https://github.com/Synthetic-Biology-Tools-for-Yeast/Yeast-Mating-Platform.

#### RNA sequencing analysis during plant infection (*F. oxysporum*).

Data acquisition was conducted and described in detail in a previous publication (52). RNA-seq data have been deposited in the Gene Expression Omnibus database (63) (Accession No. GSE243247).

### Data Analysis and Statistical Analysis.

#### Flow cytometry data and gating: NovoCyte quanteon.

All data were acquired with the NovoCyte Quanteon™ (Agilent). All flow cytometry data were extracted as FCS files and gated in FlowLogic™ v8.3 (Inivai Technologies).

#### Diploid frequency on plates.

After the quantification of the haploid strains and diploids, the limiting haploid A was determined, and Eq. **1** was applied.

[1] $$\begin{array}{c} \mathrm{Diploid}\;\mathrm{frequency}=(\mathrm{Diploids})/(\mathrm{Haploid}A+\mathrm{Diploids})*100. \end{array}$$

#### qPCR: Diploid frequency Library-on-Library.

The relative quantification of diploids was performed by comparing the cycle threshold (Ct) value of the conserved regions (R1 and R3) flanking the barcodes and the Ct value of the amplicon generated with two barcodes. All possible combinations of barcodes plus 3 technical replicates of the control (DOG77 + DOG78) were tested. The relative quantification was calculated using ∆Ct method (64, 65).

[2] $$2^{-\Delta \mathrm{CT}},$$

where ΔCT = CT (barcodes) – CT (conserved region).

## Supplementary Material

Appendix 01 (PDF)

Dataset S01 (XLSX)

Dataset S02 (XLSX)

Dataset S03 (XLSX)

## Data Availability

Next-generation sequencing data have been deposited in GitHub (https://github.com/Synthetic-Biology-Tools-for-Yeast/Yeast-Mating-Platform) (66).
