# Supplementary material for: A yeast mating platform for multiplex screening of fungal GPCR–ligand interactions
Source: Proc Natl Acad Sci U S A. 2025 Oct 24;122(43):e2521198122. doi: 10.1073/pnas.2521198122 (PMC12582325; doi:10.1073/pnas.2521198122)
Supplement: Supplementary file 1 — Appendix 01 (PDF) [file pnas.2521198122.sapp.pdf]

PNAS

Supporting Information for

A yeast mating platform for multiplex screening of fungal GPCR-ligand interactions

Giovanni Schiesaro,[1] Melani Mariscal,[4] Mathias Jönsson,[1] Ricardo Tenente,[3] Mathies B. Sørensen,[2] Marcus Wäneskog,[1] María Victoria Aguilar-Pontes,[4] Agustina Undabarrena,[1] Marcus Deichmann,[1] Emma E. Hoch-Schneider,[1] Viji Kandasamy,[1] Thomas M. Frimurer,[3] Antonio Di Pietro,[4] Line Clemmensen,[2] Michael K. Jensen,[1] Emil D. Jensen[1] *

* To whom correspondence should be addressed:
Emil D. Jensen, e-mail: emdaje@biosustain.dtu.dk

This PDF file includes:

Supporting text
Figures S1 to S13
Tables S1 to S6
Legends for Datasets S1 to S3
SI References

All materials, experimental data and statistics can be found in Supplementary Datasets 1-3.

Supplementary Figures

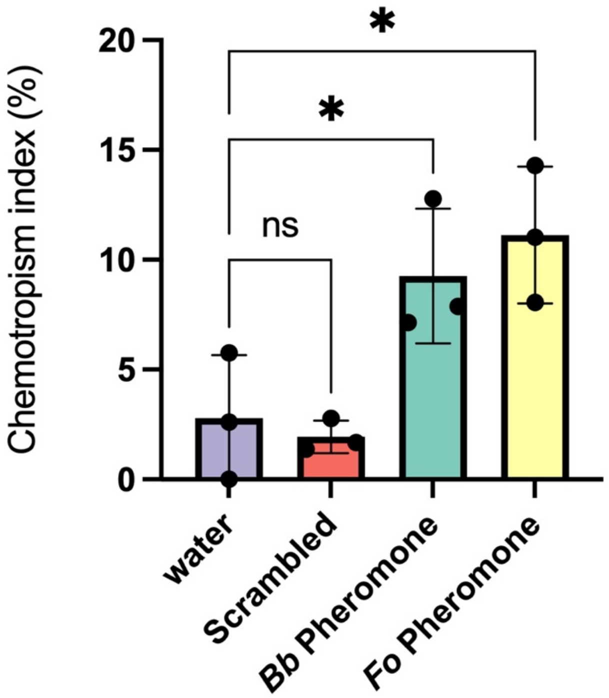

Supplementary Figure 1. Chemotropism of *B. bassiana* spores with different *Bb*, *Fo*, and a scrambled version of *Fo* pheromones. The assay was performed with 378 µM of pheromone and compared with the chemotropism induced by water. All points represent the average of three biological replicates; results represent the mean. Statistical significance was determined through one-way analysis of variance (ANOVA) with Dunnett's multiple comparisons (∗$p \leq 0.05$). *B. bassiana* chemotropism assays were conducted as previously described with a few modifications[11]. *B. bassiana* was revived in Oat Meal Agar (OMA) for a week. Spores were collected with water/glycerol 50% and stored at 4 °C. Spores were counted using a Neubauer chamber and dissolved to reach a concentration of 10^6 spores/ml. On the side glass microscope slides were sterilized and 3 lines were drawn on the back side of each slide: a scoring line in blue, a solvent line in blue, and a test compound in red. On the front side, 500 µl of water agar was added to create a thin layer in which 10 µl of test compound (378 µM of pheromone or water) and 10 µl of solvent (50% v/v methanol) were added in their respective lines. 15 µl of spores were added on the scoring line and spread with a cover glass slide. After 20 hours the germination was assessed on a Leica DM4000 B microscope (Leica Microsystems) equipped with a DFC300 FX camera (Leica Microsystems).

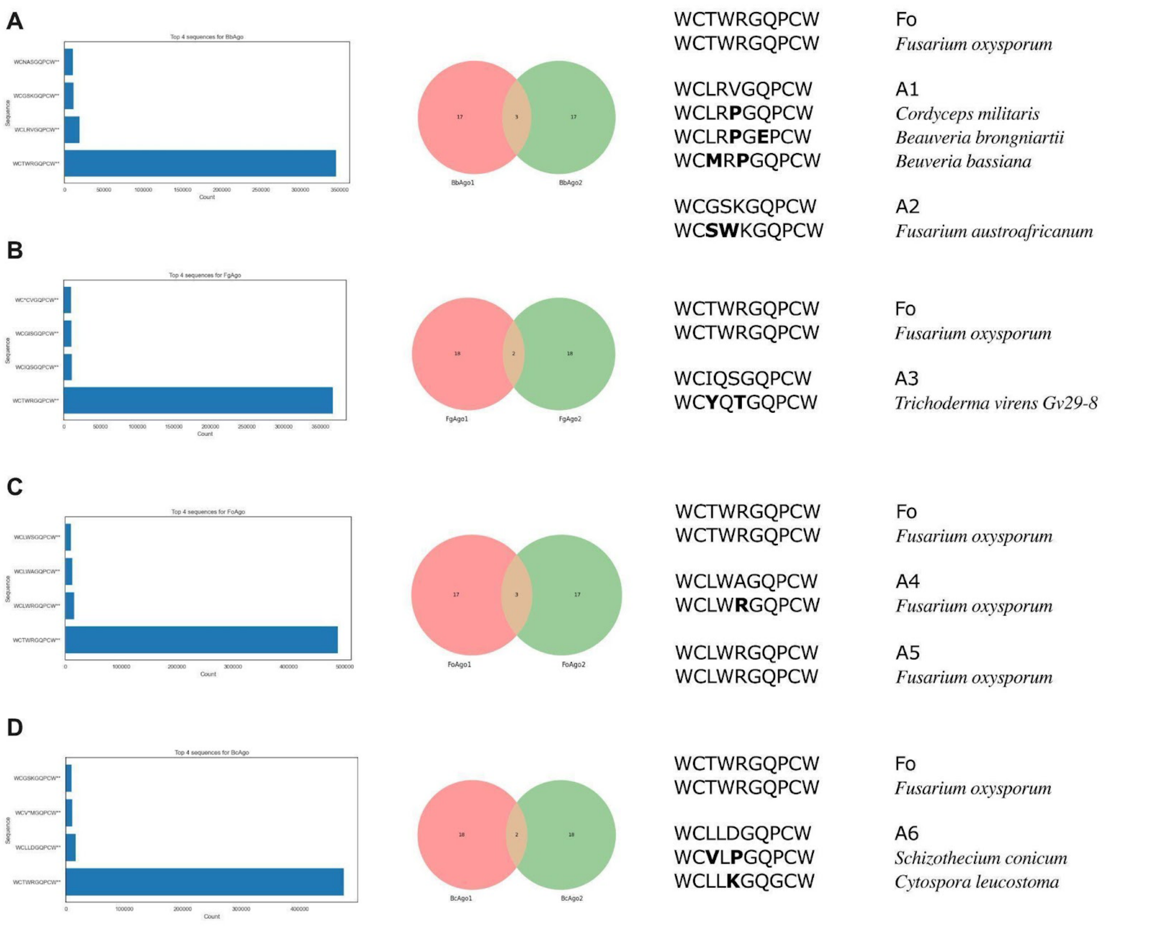

Supplementary Figure 2. Results of the pheromone library enrichment for *Bb*.Ste2, *Fg*.Ste2, *Fo*.Ste2 and *Bc*.Ste2. On the right are shown the counts of the top 4 pheromones enriched, and at the center, the Venn diagram with the intersection of the top 20 for the two biological replicates. The alignment of the most enriched pheromones found at the intersection of the two biological replicates is shown on the left. Except for *Fo* and A5, all the remaining pheromones exhibited some similarity to known fungal pheromones, but with one or two different residues.

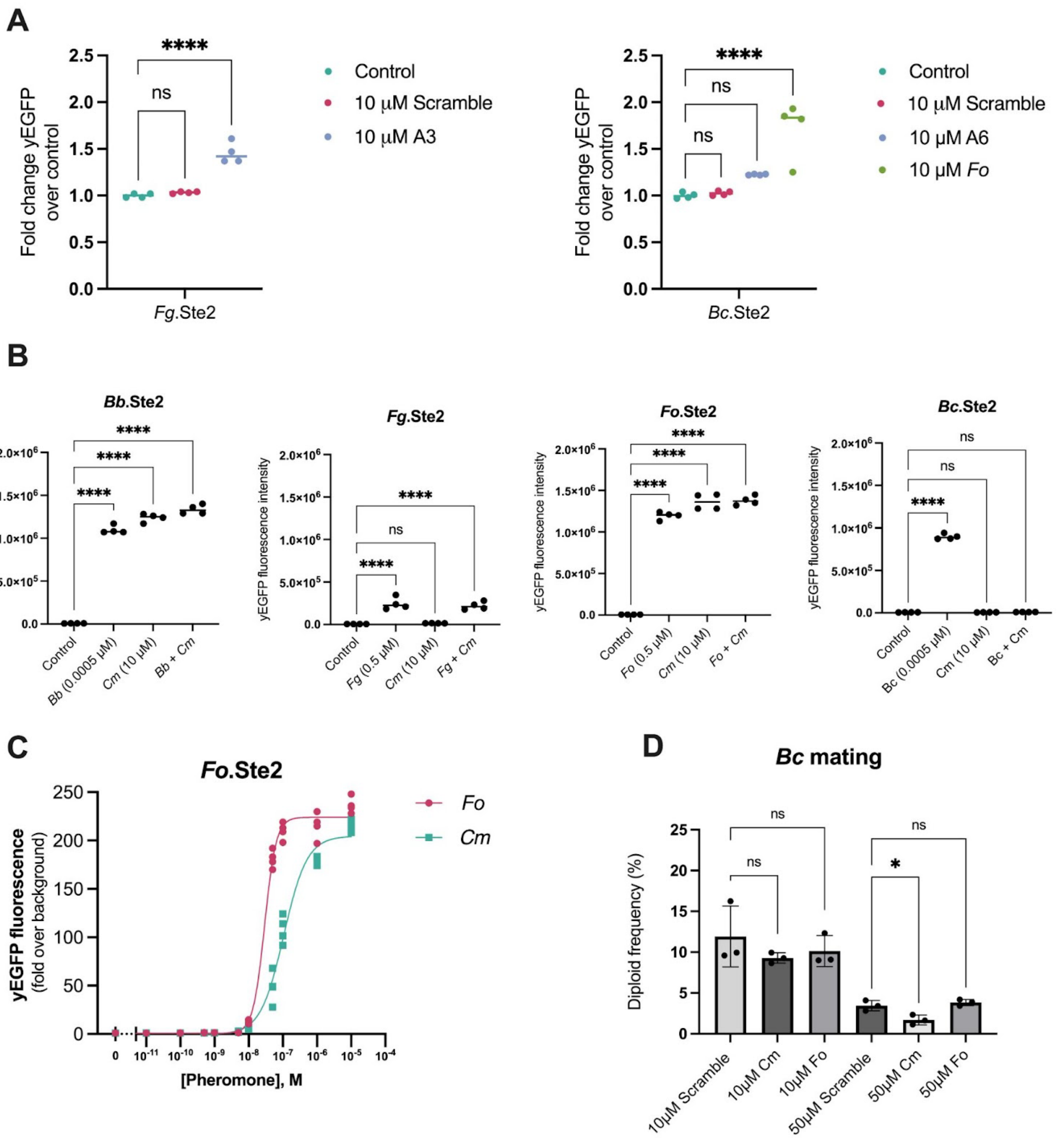

Supplementary Figure 3. A Fold-change activation of *Fg*.Ste2 and *Bc*.Ste2 with 10 μM of pheromone. **B** The effect of *Cm* pheromone incubated alone or in a mixture with the cognate agonist pheromone was compared to the control with no pheromone supplementation for *Bb*.Ste2, *Fo*.Ste2, *Fg*.Ste2 and *Bc*.Ste2 biosensor. **C** Dose-response curve of *Cm* pheromone on Fo.Ste2. **D** Effect of *Cm* Pheromone supplementation on diploid frequency formation with *Bc* strains. In **A**, **B**, and **C** means represent four biological replicates, and in **D** means and standard deviations represent the results of three biological replicates. In **A**, **B,** and **D** statistical significance was determined using one-way ANOVA with Dunnett's multiple comparison test in GraphPad Prism (✱$p \leq 0.05$,✱✱$p \leq 0.01$, ✱✱✱ $p \leq 0.001$, ✱✱✱✱$p \leq 0.0001$).

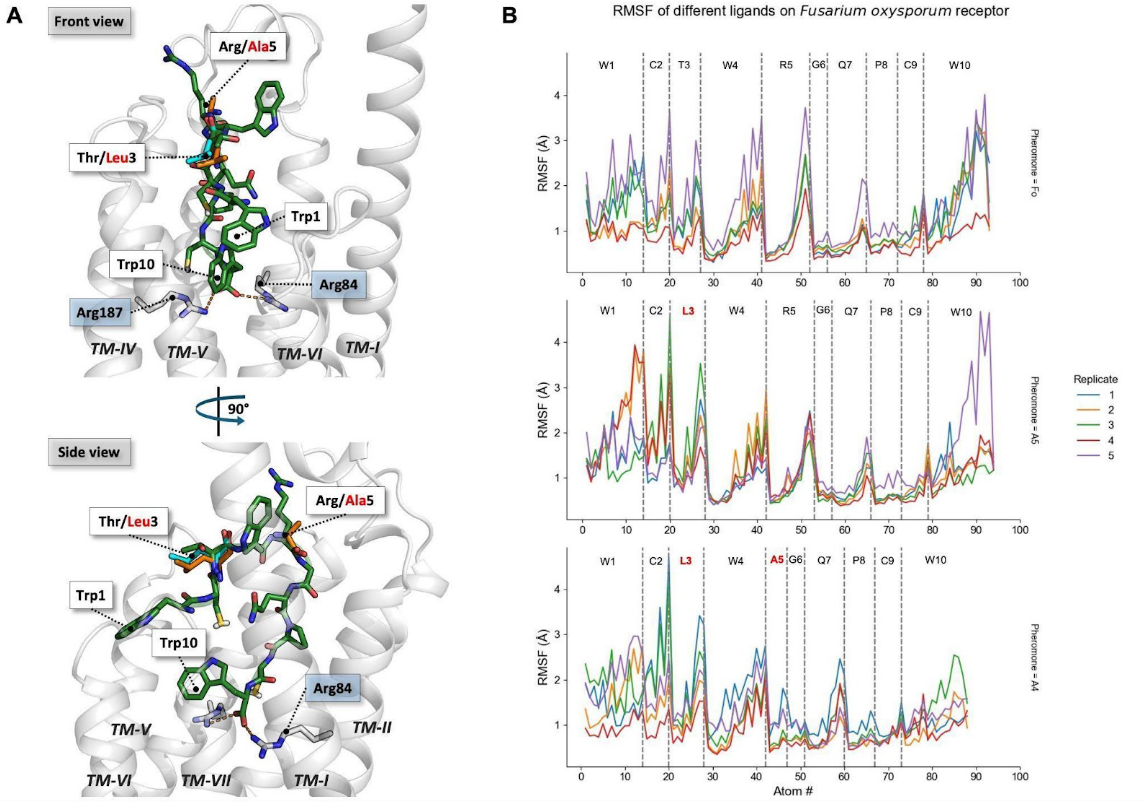

Supplementary Figure 4. A Proposed Binding pose of *Fo* pheromone (green) in *Fo*.Ste2. Arg84 and Arg187 interact with the carboxylic acid functional group of Trp10. A5 (cyan) and A4 (orange) pheromones deviate from the *Fo* pheromone by 1 and 2 residues, respectively. **B** Root-mean-square-fluctuation (RMSF) of non-hydrogen atoms of *Fo*, A5, and A4 pheromones inside *Fo*.Ste2 pocket. Dashed vertical lines separate the pheromone residues. Residues of A5 and A4 pheromones highlighted in red deviate from the *Fo* pheromone.

GPCR and ligand complexes were predicted with AlphaFold2 Multimer[1] using ColabFold[2]. Receptor-ligand complexes predicted in a similar pose to *Sc*.Ste2 receptor-ligand complexes[3] were selected. Protein preparation and minimization, system setup, MD simulations, and data analysis were performed using Maestro (Schrödinger Release 2024-1). GPCRs were restricted to residues in the N-terminal and the transmembrane domains (1-291). The force field of the ligands was generated with LigPrep. GPCR-ligand complexes were prepared with the Protein Preparation Wizard[4] at pH 5.6, which included capping GPCR chain termini with neutral acetyl and methylamide groups, assignment of histidine protonation states, and whole structure minimization. *F. oxysporum* pheromone does not establish intramolecular disulfide bonds[5]; therefore, the intramolecular disulfide bond was reduced for all ligands. The System Builder module was used to prepare the

orthorhombic simulation box (10x10x10 Å), with the prepared GPCR-ligand complex embedded in a pre-equilibrated membrane (300K) palmitoyl-oleoyl-phosphatidylcholine (POPC) bilayer aligned with PPM3.0 of the OPM database alignment[6], TIP3 solvent neutralized with 0.15 Na+/Cl- ions, and the OPLS4 force field[7]. Membrane model system default relaxation was used to minimize the system, and the final snapshot was used to run five independent production runs with random seeds for each GPCR-ligand complex using Desmond[8]. Simulations were run at 300 K for 300 ns and snapshots were stored at every 500-ps interval.

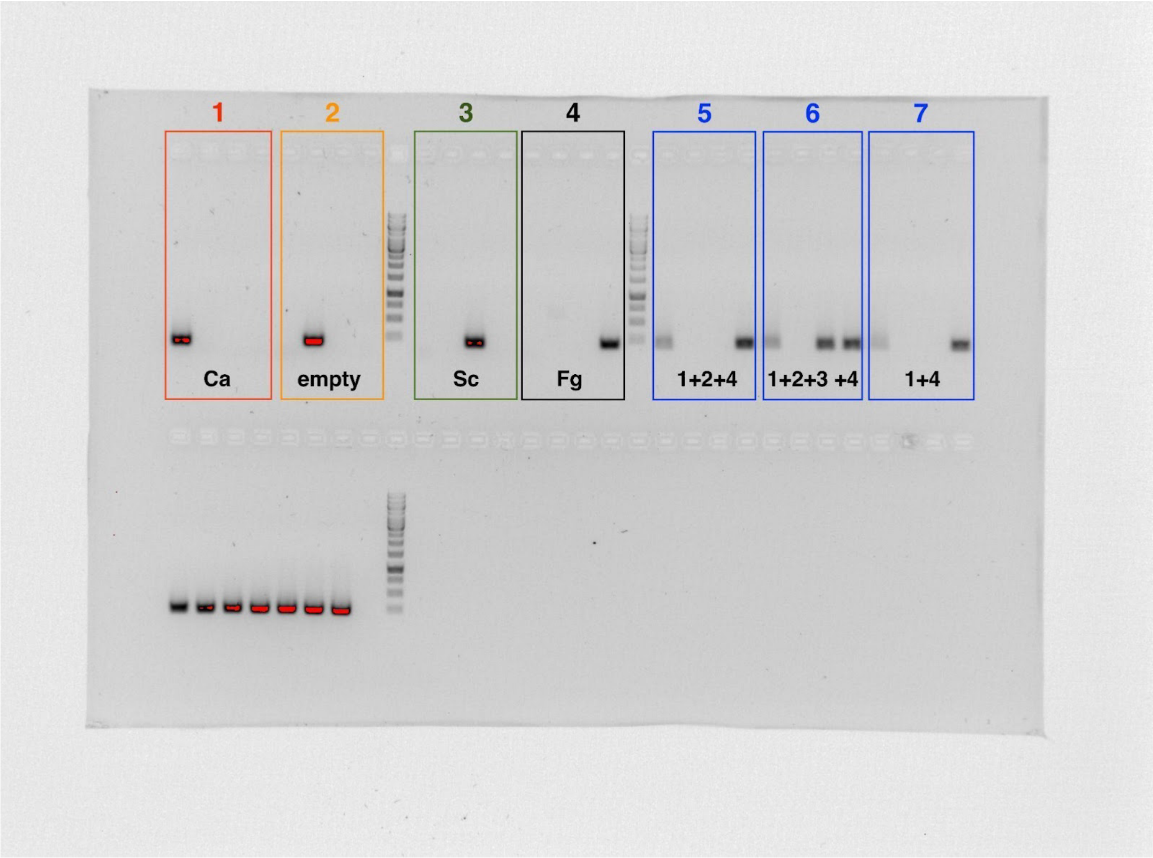

Supplementary Figure 5. Co-cultures (boxes 1, 2, 3, and 4) of the *Ca* GPCR and pheromone strains (GEN101 + GEN102), empty pair (GEN104 + GEN105), *Sc* pair (GEN106 + 107), and *Fg* pair (GEN108+ GEN109) with a specific amplification for their barcodes combination (in order *Ca*, empty, *Sc*, *Fg* primers). Consortia of different co-cultures of strains (boxes 5, 6, and 7). Box 5 contained the *Ca* + empty + *Fg* co-cultures, box 6 contained all four co-cultures, and box 7 contained the *Ca* + *Fg*. On the bottom, all co-cultures or consortia (1 to 7) had the R1 and R3 conserved regions flanking the two barcodes.

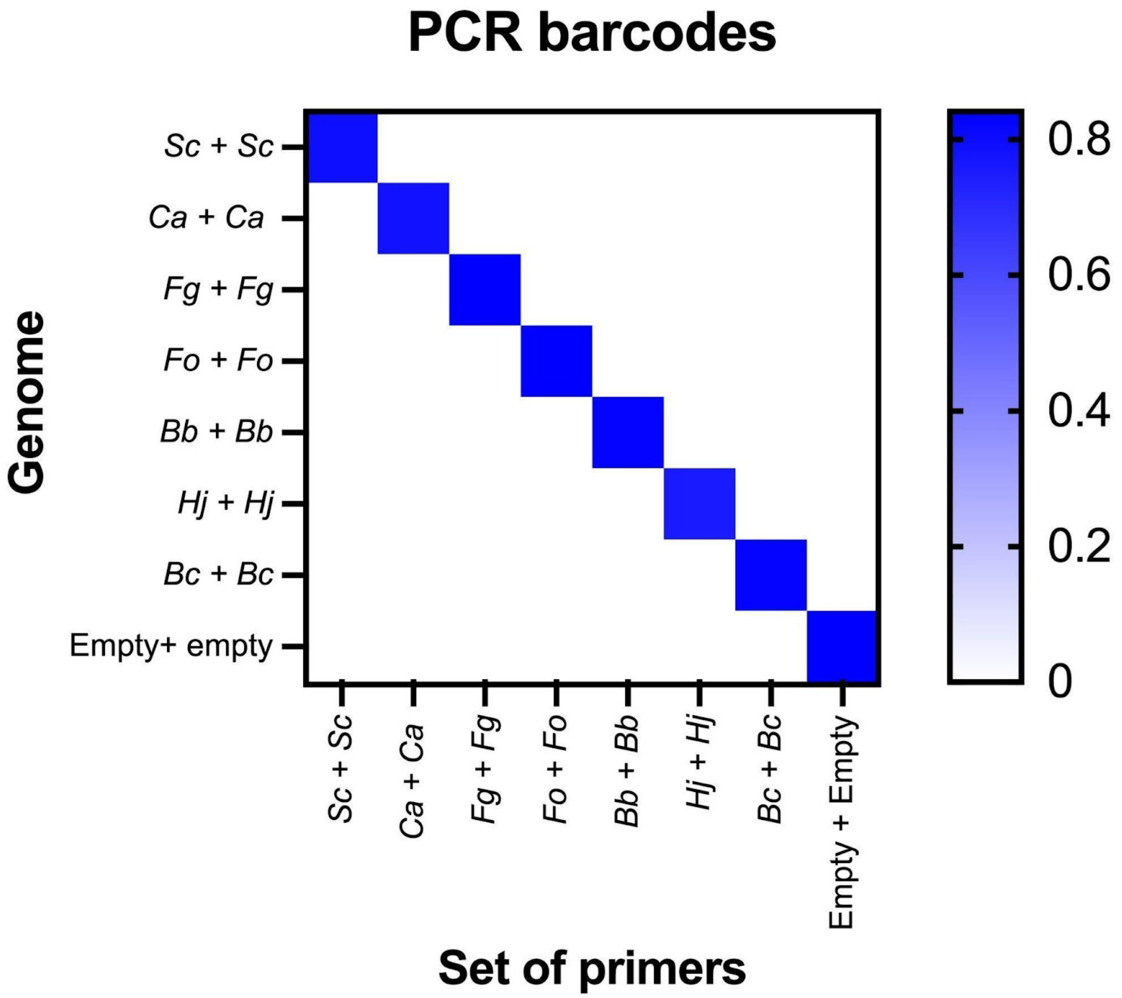

Supplementary Figure 6. After co-culture, the genome of the diploid cells containing two specific barcodes was used as a template for different sets of primers. We didn't observe any unspecific amplification. The proportion of diploid cells detected with different primer sets was consistent in all co-cultures (Supplementary Data 2-3).

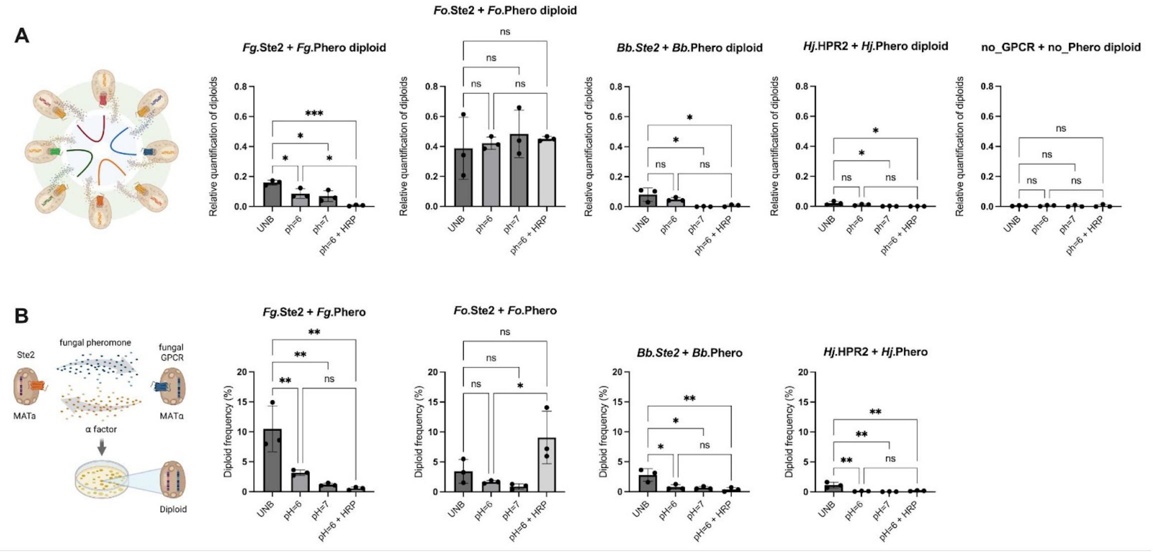

Supplementary Figure 7. Comparison between **A** the consortia with *Fg*, *Fo*, *Hj*, *Bb* GPCRs, and pheromone strains by adding two negative controls (empty strains) in liquid cultures (GEN108 + GEN109 + GEN114 + GEN115 + GEN110 + GEN111 + GEN116 + GEN117 + GEN104 + GEN105) and **B** the co-culture of *Fg* (GEN108 + GEN109), *Fo* (GEN114 + GEN115), *Bb* (GEN110 + GEN111) and *Hj* (GEN116 + GEN117) strains on plates. Means and standard deviations represent the results of three biological replicates. Statistical significance was determined using one-way ANOVA with Tukey's multiple comparison test in GraphPad Prism (∗$p \leq 0.05$,∗∗$p \leq 0.01$, ∗∗∗ $p \leq 0.001$).

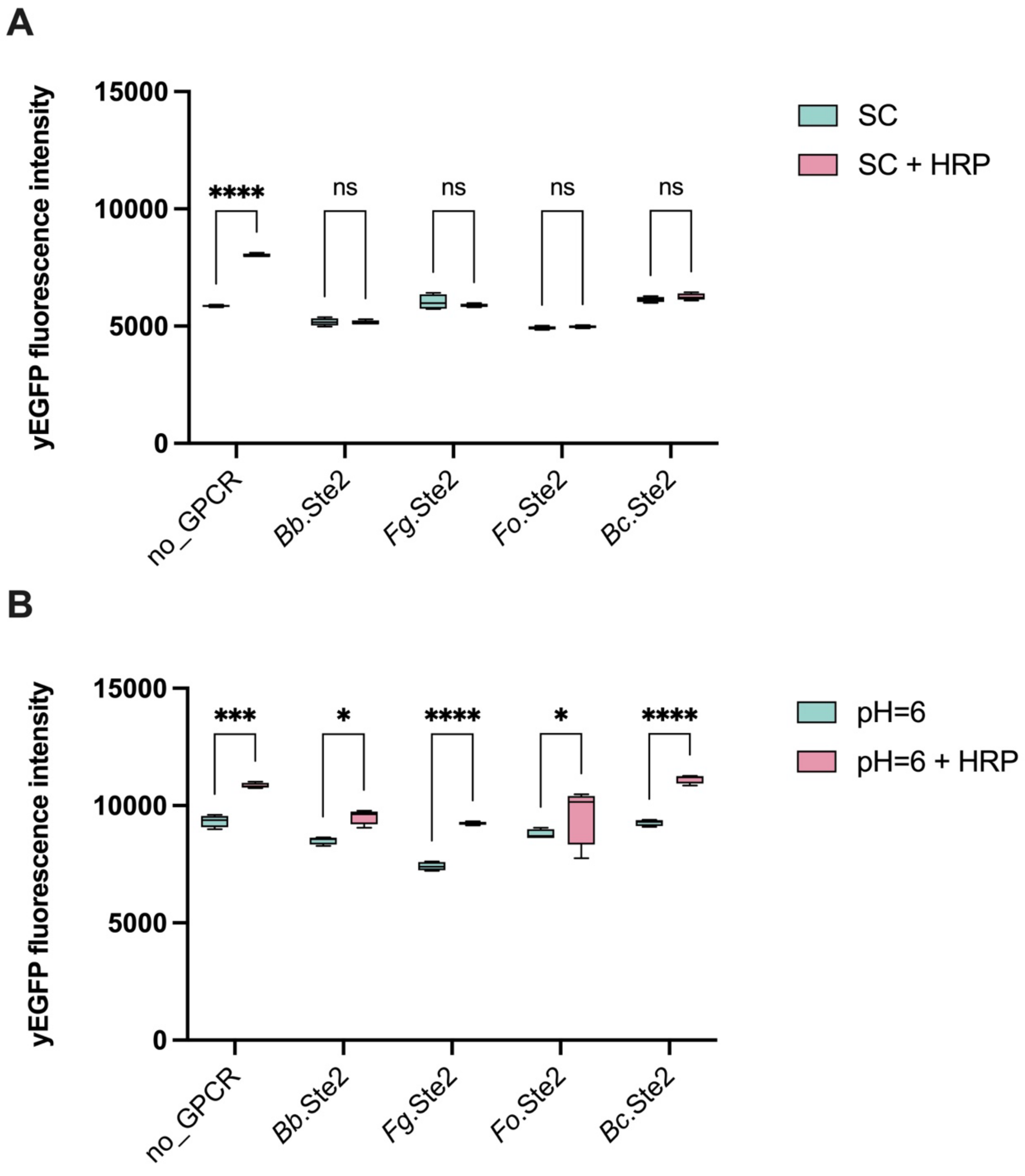

Supplementary Figure 8. Effect of HRP on yeast biosensors. **A** Activation of the pheromone response pathway was only observed in the background strain without a GPCR in SC media. **B** A general activation trend was observed in SC-AS/Urea pH=6 + 2 μM in all the biosensors *Bb*.Ste2 (GEN88), *Fg*.Ste2 (GEN87), *Fo*.Ste2 (GEN90) and *Bc*.Ste2 (GEN89) and the background control strain with no GPCR (CPK423). Statistical significance was determined using two-way ANOVA with Tukey's multiple comparison tests in GraphPad Prism (✱$p \leq 0.05$,✱✱✱$p \leq 0.001$, ✱✱✱✱$p \leq 0.0001$).

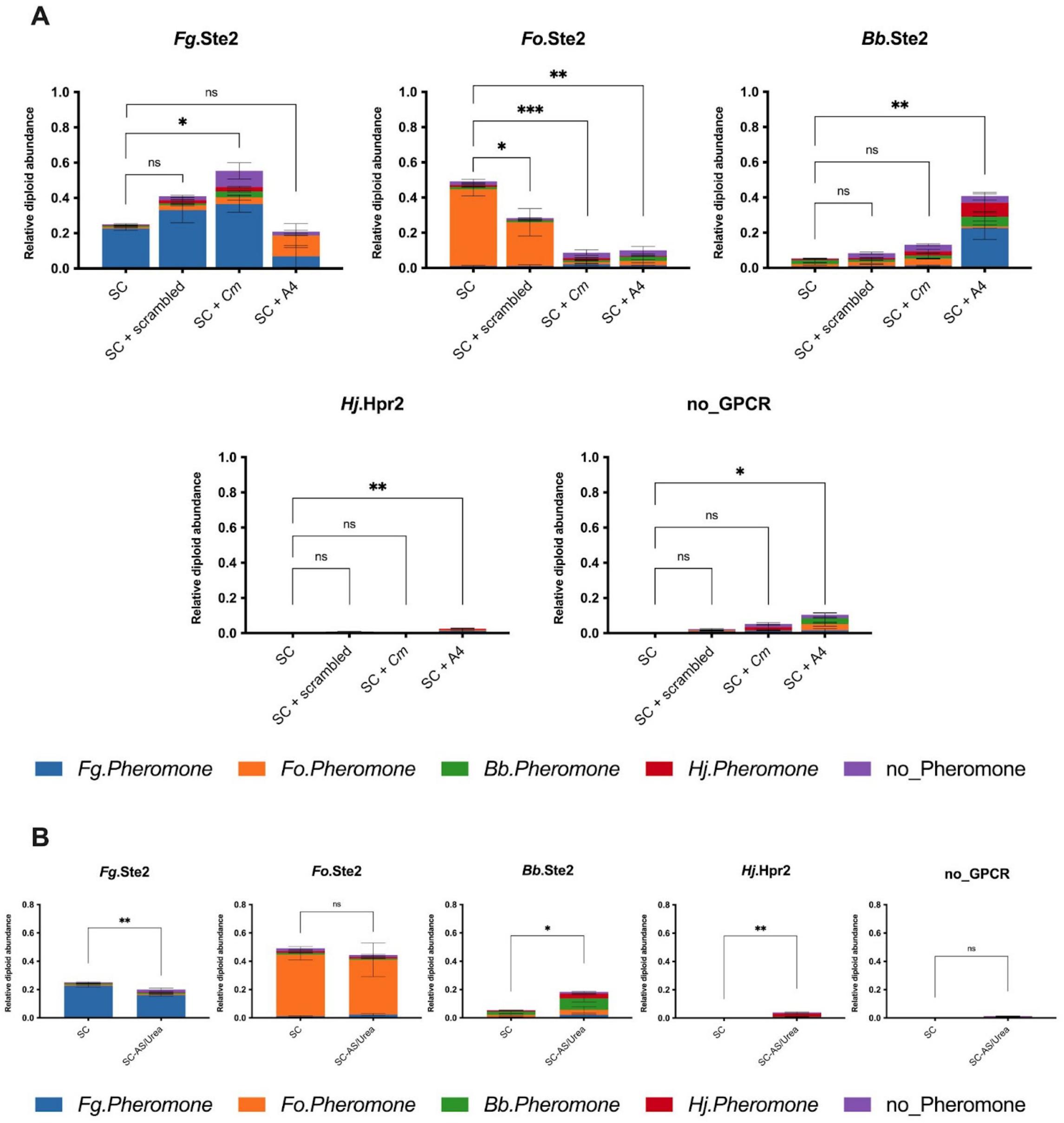

Supplementary Figure 9. A-B Relative diploid abundance for each fungal GPCR from consortia of *Fg*, *Fo*, *Hj*, *Bb*, negative control GPCRs, and pheromone strains (GEN108 + GEN109 + GEN114 + GEN115 + GEN110 + GEN111 + GEN116 + GEN117 + GEN104 + GEN105). **A** Diploid distribution in SC media without (SC) and with different supplementation of pheromones (SC + scrambled, SC + *Cm,* or SC + A4). All pheromones were supplemented at 10 μM. **B** Comparison of the relative diploid abundance between SC with ammonium sulfate (SC) and SC with ammonium sulfate and Urea (SC-AS/Urea). Statistical significance plotted on graphs was determined using

one-way ANOVA with Dunnett's multiple comparison test in GraphPad Prism (∗$p \leq 0.05$,∗∗$p \leq 0.01$, ∗∗∗$p \leq 0.001$) in **A,** while unpaired t-test (∗$p \leq 0.05$, ∗∗$p \leq 0.005$) was used in **B**.

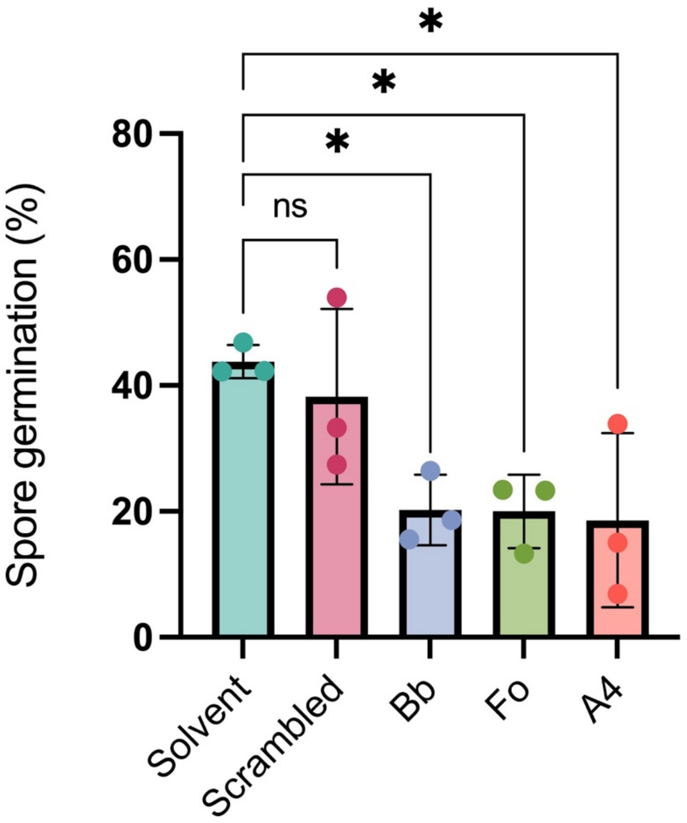

Supplementary Figure 10. Germination of *F. oxysporum* microconidia after 13 h of incubation in Germination Media at a cell density of 3.2 x 10^6 microconidia/mL. The assay was performed with 400 µM of pheromone. All points represent the average of three biological replicates in which at least 300 spores were counted. Statistical significance was determined through one-way analysis of variance (ANOVA) with Dunnett's multiple comparisons (∗$p \leq 0.05$).

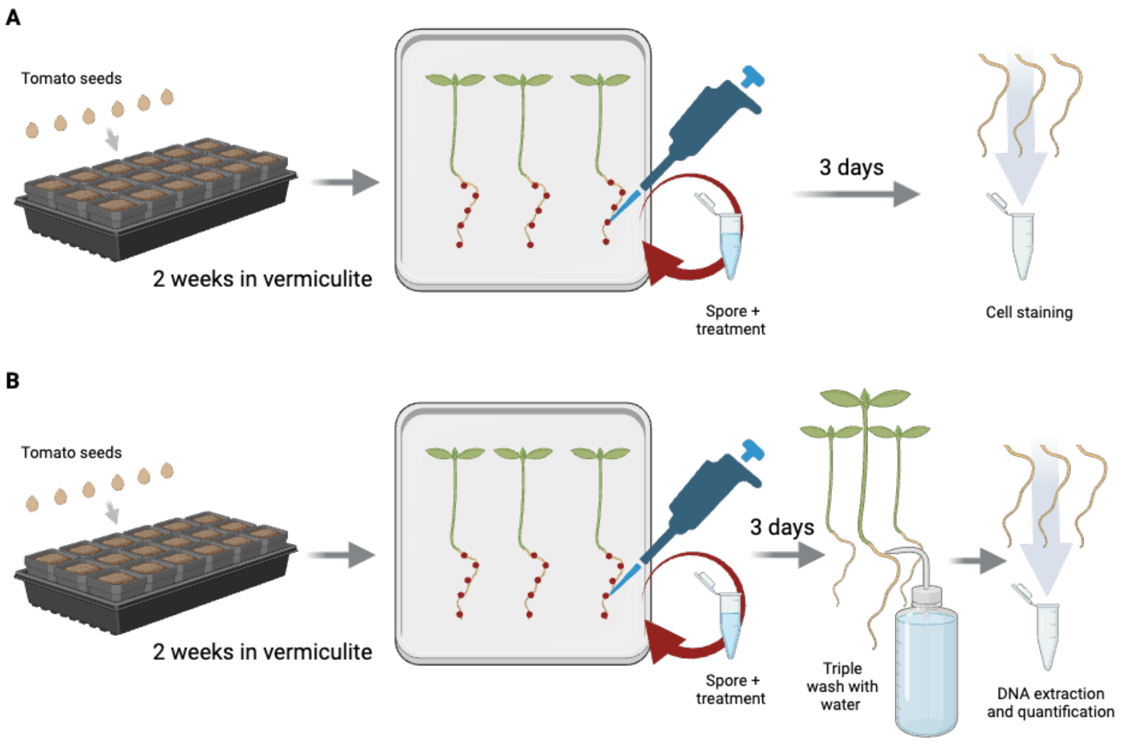

Supplementary Figure 11. Representation of the plant infection assay for **A** fluorescence microscopy of *F. oxysporum* infection, and **B** the relative quantification of fungal biomass in tomato roots.

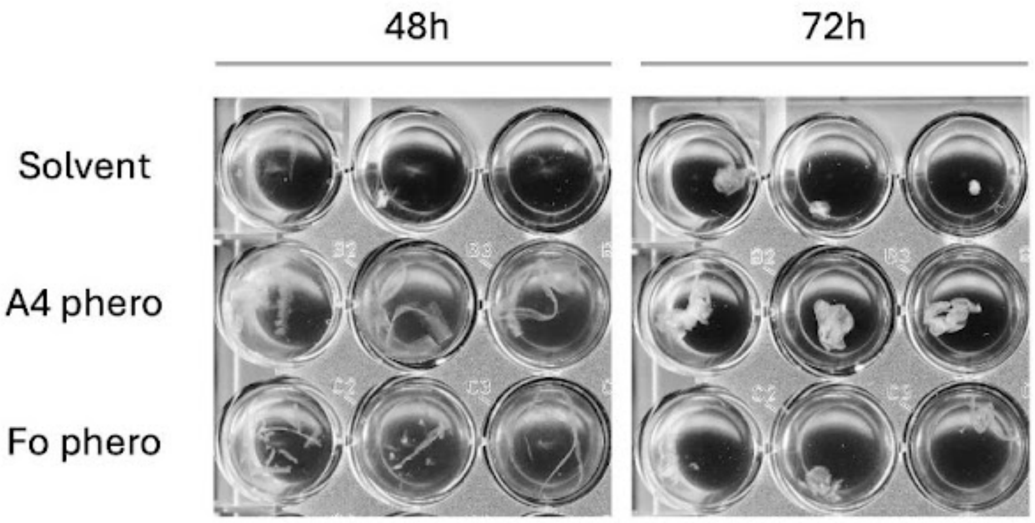

Supplementary Figure 12. Formation of hyphal aggregates after 48 and 72 hours in the plant root exudate media. The microtiter plate with a cell density of 2.5 x 10^6 microconidia/mL was maintained at 28℃ and 170 rpm.

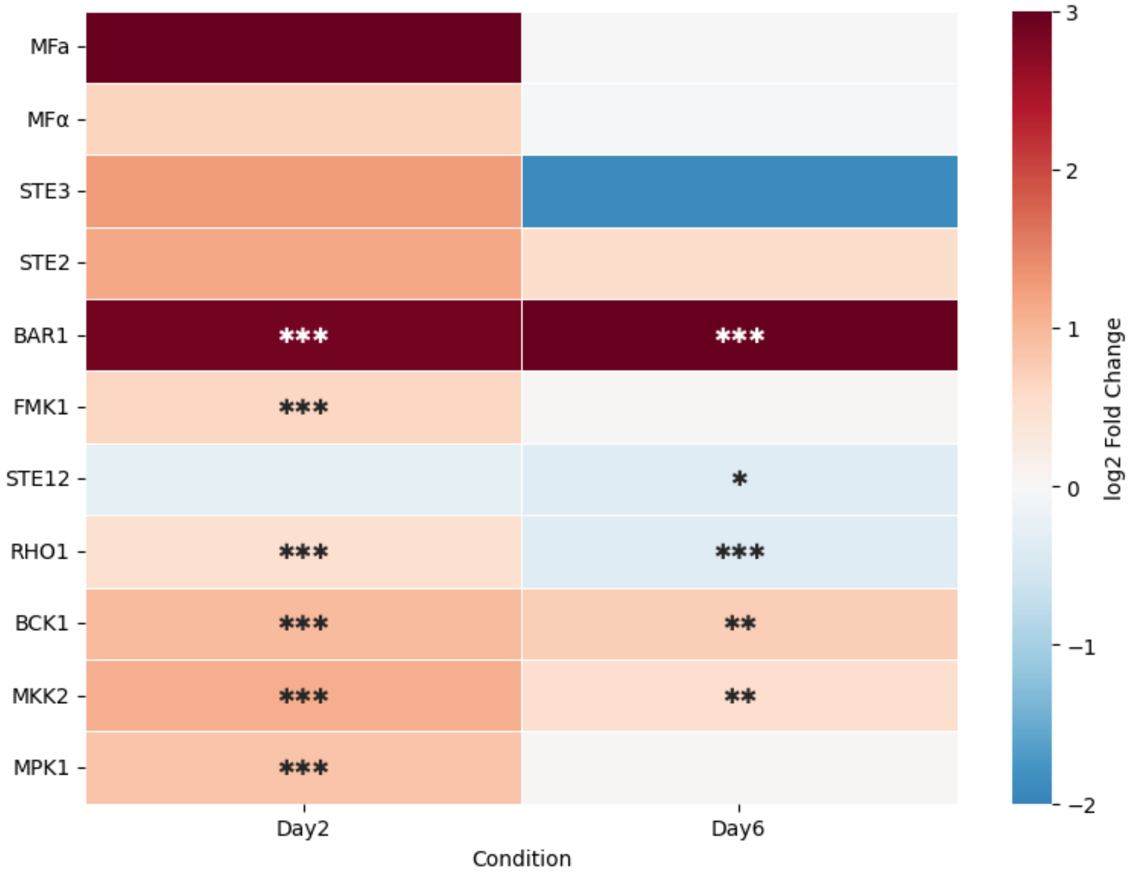

Supplementary Figure 13. Heatmap showing the relative expression in *F. oxysporum* after 2 and 6 days of tomato plant infection. The selected genes include pheromones, mating receptors, BAR1, and those involved in the MAPK pheromone response pathway and the cell wall integrity (CWI) MAPK cascade. The fold-change and the p-value were calculated with DESeq2 (∗$p \leq 0.05$, ∗∗p≤ 0.01, ∗∗∗p≤ 0.001).

Supplementary Tables

Supplementary Table 1 - Fungal species and pheromones

Pheromone name	Organism	AA Sequence
Sc	*Saccharomyces cerevisiae*	WHWLQLKPGQPMY
Ca	*Candida albicans*	GFRLTNFGYFEPG
Fg	*Fusarium graminearum*	WCWWKGQPCW
Fo	*Fusarium oxysporum*	WCTWRGQPCW
Bb	*Beauvaria bassiana*	WCMRPGQPCW
Hj	*Hypocrea jecorina (Trichoderma reseei)*	WCYRIGEPCW
Bc	*Botrytis cinerea*	WCGRPGQPC
Nc	*Neurospora crassa*	QWCRIHGQSCW
Mo	*Magnaporthe oryzae*	QWCPRRGQPCW

Supplementary Table 2 - Pheromone tested

Pheromone name	Description	AA Sequence
A1	Bb.Ste2 - Agonist candidate	WCLRVGQPCW
A2	Bb.Ste2 - Agonist candidate	WCGSKGQPCW
A3	Fg.Ste2 - Agonist candidate	WCIQSGQPCW
A4	Fo.Ste2 - Agonist candidate	WCLWAGQPCW
A5	Fo.Ste2 - Agonist candidate	WCLWRGQPCW
A6	Bc.Ste2 - Agonist candidate	WCLLDGQPCW
Cm	from *Cordycept militaris*	WCLRPGQPCW
Scrambled	Scrambled version of *F. oxysporum* pheromone	WRWPCCWGQT

Supplementary Table 3 - DNA amplicon sequence of YLP1

ACTACTATTGCCAGCATTGCTGCTAAAGAAGAAGGGGTATCTCTCGATAAAAGAGAGGCT
GAAGCT**TGGTGCNNNNNNNNNNGGACAACCTTGTTGG**TAATAGAGGACAGATGTAGATAC
GTTGTTGACACTTCTAAATAAGCGAATTTCTTATGATTTATGATTTTTATTATTAAATAAGTT
ATAAAAAAAATAAGTGTATACAAATTTTAAAGTGACTCTTAGGTTTTAAAACGAAAATTCTTA
TTCTTGAGTAACTCTTTCCTGTAGGTCAGGTTGCTTTCTCAGGTAT

Supplementary Table 4 - Pheromone genes

Specie	Annotation	seq
Fusarium oxysporum f. sp. lycopersici 4287	FOXG_08636	MKYSFVTLAAVAGAALAAPPPSAIDNFGPDFFTPFCNLDYKGKPCEELVGKGDKNADAICKAGREHFCGPQKRDAVPEPQPDPVADPMPWCTWRGQPCWKEKMKAKREAEAIPEPIAAPQPDPVADPMPWCTWRGQPCWKEKMAKREAIPEPVAAPQPDPVADPMPWCTWRGQPCWKEKMRMAKREPEPVAAPQPDPVAEPMPWCTWRGQPCWKKTKRAAAPEPAPEAENEPRWCLWRGQPCWKKTKRDATPEPWCLWRGQPCWKAKRDAAPEPWCMWRGQPCWKAKRDAGQALSNALHATRSLDTRSADAPSTAHLPRDAAHKAKRSIVELANLIALSARGSPEEYFKSLELETFFPDAAPNATAKRDLNTLQEDKRWCMWRGQPCWKAKRAAEAVLDAVDGDDGATGPGGPDSHYDTRDFKSENFAAKRDLIAIKAAARSIADMSEE
Beauveria bassiana ARSEF 2860	pheromone precursor	MKLSLVMLATAATTVIAAPRPWCMRPGQPCWKLKRAVDALGEPAPSPVEPLDADNIGLFASGAHDRLLHLASSDAANVDDEGAFEKRWCMQTPKCWKLLADEDGELSKRWCMRPGQPCWKRSVDEHGDLAKRWCMRPGQPCWKAKRAAESVLNAGQEDGDAQEQDCGDDGECSVAKRHLDGLHHVARAIVEAF
Beauveria asiatica	hypothetical protein G3M48_002203	MKLSLVMLATAATTVLAAPRPWCLRPGQPCWKLKRAVDVLGEPASTPSPVDNNAVPAVGADNLGLFASGAHDRLLHLAAQASYDPADADEAAAFERRWCTQNPKCWKRNVDEHGQLAKRWCLRPGEPCWKRGVDEHGDLAKRWCLRPGEPCWKAKRAAESVLNAGQEDGDAQEQDCGDDGECSVAKRHLDGLHHVARAIVEAF
Cordyceps militaris	clock-controlled pheromone ccg-4 precursor	MKFEFAALLALAATGLAAPSPWCTRPGQPCWKLKRAVEAVDAHADAEAAADGMGLIASVAYDRLVELAAQGSPDPAAFYEQHRLQKSKRDVEAAAAIEKRWCTRPGEPCWKRAVEEQDELSKRWCLRPGQPCWKAKRAAESVLEAGQEDSTEECGEGDEQCSNAKRSLENLHQVARAIVEAF
Fusarium graminearum	CAF3480000.1	MKYSILTLAAVASTTLAVAVPAPQPDPVAEPMPWCTWKGQPCWKEKMARREAQPEPEAVAAPEPDPVAEPMPWCTWKGQPCWKEKMAKRAAKPEPVPAPQPDPVAEAEPWCTWKGQPCWKEKMVKRAAEAEAEAEPIPDPVAAPQPDPVAEPMPWCTWKGQPCWKEKMAKREAKPEPWCWWKGQPCWKAKRDAAPEPWCWWKGQPCWKAKRNAAPEPMPEPANEPRWCWWKGQPCWKSKSKRDASPEPWCWWKGQPCWKAKRDAAPEPWCWWKGQPCWKAKRNAAPEPMPEPANEPRWCWWKGQPCWKSKSKRDASPEPWCWWKGQPCWKAKRDAGEALTVALHATRGVETRSVAETEHLPRDAAHQAKRSIVELANVIALSARGSPEEYFKHLYLEEFFPEIPHNATAKRDVKTLQEDKRWCWWKGQPCWKAKRAAEAVLHAVDGSDGAGAPGGPEEHFDTSHFNPQNFEAKRDLMAIKAAARSVVESLEG

Botrytis cinerea B05.10	hypothetical protein BCIN_01g07500	MKFTNAIALAILAATATAVAVPEPWCGRPGQPCKREAVAVAAPVAEPWCGRPGQPCKRTPEAEAWCGRPGQPCKRDAEPWCGRPGQPCKREALPEAWCGRPGQPCKRTPLAEAEAEAWCGRPGQPCRKNKRAAEAVAEAFAEPWCGRPGQPCKRDAEADVSEAAIKRCNMVGGACFEAKRLARDLAEATAETVEDSDLFLRSLNIETREVSEVVAREAEAWCGRPGQPCKRDAEAWCGRPGQPCKREALAEAEAWCGRPGQPCKREALAEAEAWCGRPGQPCKRTAEPWCGRPGQPCKEKREADPEAEAWCGRPGQPCRAVKRAAEAIAEALAEPTAEAWCGRPGQPCKREALAEAEANAEAWCGRPGQPCRKAKRDAFALAYAADVALAQL

Supplementary Table 5 - Barcode system

MATα
cccaaagctaagagtcccattttattcttctatatgtatattttcgatactctaaaccaccctacaatgtagccctatactaaatctgctcaatttcagcttctacaagtgactcgaggccacgtggaaagatccaactactccagcacaacgattcaatataatcgattgctccactataagaggcaagaacaagcttcaacttttggtaagccgccgtttataaacagggaagatgtcctttgtcaagggaggcacagagcatggccaatttggcaaattgcaggtttttctgagtgaaaaatgaaaaagcattgtagtagagtcggctcactgaaaaaccggggaggacgaaaaggtttccagccacagttgtagtcacgtgcgcgccatgctgactaatggcagccgtcgttgggcagaagagaattagtatggtacaggatacgctaattgcgctccaactaccaaggttgttgagggaacactggggcaataggctgtcgccattcaagagcagattaagtcctcagcgagctcgcatggaatgcgtgcgatAGTGCGATCCACCCTCCCATTAGTCCGCNNNNNNNNNNNNNNNNNNNNCGGAGATTACCGAGCAATCTATCAGATCGGCGACCATTAGTGAGCTACTGGAGCCGAGGGATAACTTCGTATAGCATACATTATACGAACGGTAACCGCGTTatcgcgtacccaattcgccctatagtgagtcgtattacgcgcgctcactggccgtcgttttacaacgtcgtgactgggaaaaccctggcgttacccctgcaggactagtgctgaggcattaatacatgggaagattcgcttttttttttttgaattacaatagtatgtctgatgtctgcaagaagtaacaggcgtgtgcacaagaatacgtgtgtgtgcgtaagcgtatgcactggtggcataacttatctaagaagtatatatcactgacatagaaatgtagatatacaggtatttttctcgataatcgataaaaatctcgtcgcgctgaaccaaacttggtggttacggagagtttttctctcatcattactgtctttcgcattgatttcccctttgaccgataaaatcccttggattcataagattaaacaaagaggtgatcaaagagaaccctgtgaaagtttatgtttataaccgggcataaagtgaactagacactttcaagaagccaaccaaagcatgagtaacgaagcttaccagcatgatcataccgtaaatcctcaccag
MATa
cccaaagctaagagtcccattttattcttctatatgtatattttcgatactctaaaccaccctacaatgtagccctatactaaatctgctcaatttcagcttctacaagtgactcgaggccacgtggaaagatccaactactccagcacaacgattcaatataatcgattgctccactataagaggcaagaacaagcttcaacttttggtaagccgccgtttataaacagggaagatgtcctttgtcaagggaggcacagagcatggccaatttggcaaattgcaggtttttctgagtgaaaaatgaaaaagcattgtagtagagtcggctcactgaaaaaccggggaggacgaaaaggtttccagccacagttgtagtcacgtgcgcgccatgctgactaatggcagccgtcgttgggcagaagagaattagtatggtacaggatacgctaattgcgctccaactaccaaggttgttgagggaacactggggcaataggctgtcgccattcaagagcagattaagtcctcagcgagctcgcatggaatgcgtgcgatAGTGCGATTACCGTTCGTATAGCATACATTATACGAAGTTATTCGTCGCTAGCGATTACACTCCAGTCTCATTATAATCGTTCGCTATTCAGGGATTGACCANNNNNNNNNNNNNNNNNNNNGATGATAGTCATGAGTGCCGACCGCGTTatcgcgtacccaattcgccctatagtgagtcgtattacgcgcgctcactggccgtcgttttacaacgtcgtgactgggaaaaccctggcgttacccctgcaggactagtgctgaggcattaatacatgggaagattcgcttttttttttttgaattacaatagtatgtctgatgtctgcaagaagtaacaggcgtgtgcacaagaatacgtgtgtgtgcgtaagcgtatgcactggtggcataacttatctaagaagtatatatcactgacatagaaatgtagatatacaggtatttttctcgataatcgataaaaatctcgtcgcgctgaaccaaacttggtggttacggagagtttttctctcatcattactgtctttcgcattgatttcccctttgaccgataaaatcccttggattcataagattaaacaaagaggtgatcaaagagaaccctgtgaaagtttatgtttataaccgggcataaagtgaactagacactttcaagaagccaaccaaagcatgagtaacgaagcttaccagcatgatcataccgtaaatcctcaccag

In blue the X-4 UP and X-4 DOWN sites. In red is the R1 sequence, in orange is the R3 sequence, in green is the barcode sequence, and in pink are the two Crelow sites (Lox 66 in MATα and Lox 71 in MATa).

Supplementary Table 6 - barcode sequences

Name	seq	strain
R1-B01 empty MATα	GCAGCTGCTACATATCGCCT	GEN104
B02-R3 empty MATa	AACTACAGTGCCGCTTACAG	GEN105
R1-B03 wt MATα	CACGCGTAGTGAGACTTACA	GEN106
B04-R3 wt MATa	TCGCTGAGCTAATAGTTGCC	GEN107
R1-B05 Ca MATα	CTCGGACCTGTGACGACCAA	GEN101
B06-R3 Ca MATa	CCTTGTCTATCCAGATTGAC	GEN102
R1-B07 Fg MATα	GGTATCGTGCATGTAGCGTA	GEN108
B08-R3 Fg MATa	TATGAACAACGCAAGGATCG	GEN109
R1-B09 Bb MATα	GAGCCCATGGTTGAATGACT	GEN110
B10-R3 Bb MATa	TGGATGGAAGAGGTACGGAC	GEN111
R1-B11 Bb MATα	CTGACTTGAGTGCTCCAAGC	GEN112
B12-R3 Bc MATa	GAAGTGTCTACTGTCCCTAA	GEN113
R1-B13 Fo MATα	ACACGCGTCACCTTAGTTGT	GEN114
B14-R3 Fo MATa	TAGCGTCGAGCTCGTTGAAC	GEN115
R1-B15 Hj MATα	GAGTAAGTGCTCCTGAGGTT	GEN116
B16-R3 Hj MATa	AGCTCCGGCAAGCAATTAAG	GEN117

Dataset S1 (separate file).

- Supplementary Data 1: contains all strains, plasmids, oligos
- Supplemementary data 2: contains all data of the experiements
- Supplemementary data 3: contains all statistical analysis

  Data analysis, statistical analysis, and graphing were done in GraphPad Prism v10 (GraphPad Software) and Jupyter Notebook (Supplementary Data 3). Sigmoidal dose-response curve fits were computed using nonlinear regression by the variable slope (four parameters) model in GraphPad Prism v10. The significance of the experiments was assessed by one-way or two-way ANOVA multivariate test and post-hoc analysis by Dunnett's or Tukey's multiple comparison test, respectively. All statistical tests were done in GraphPad Prism v10 (GraphPad Software) with a default 95% confidence interval ($\alpha = 0.05$) applied and multiplicity-adjusted *p* values were reported to account for all multiple comparisons within tests.

SI References

1. Vans, R. *Protein Complex Prediction with AlphaFold-Multimer*. (2021). doi:10.1101/2021.10.04.463034.
2. Mirdita, M. *et al.* ColabFold: making protein folding accessible to all. *Nat. Methods* **19**, 679–682 (2022).
3. Velazhahan, V., Ma, N., Vaidehi, N. & Tate, C. G. Activation mechanism of the class D fungal GPCR dimer Ste2. *Nature* **603**, 743–748 (2022).
4. Sastry, G. M., Adzhigirey, M., Day, T., Annabhimoju, R. & Sherman, W. Protein and ligand preparation: parameters, protocols, and influence on virtual screening enrichments. *J. Comput. Aided Mol. Des.* **27**, 221–234 (2013).
5. Vitale, S. *et al.* Structure-activity relationship of α mating pheromone from the fungal pathogen Fusarium oxysporum. *J. Biol. Chem.* **292**, 3591–3602 (2017).
6. Lomize, A. L., Todd, S. C. & Pogozheva, I. D. Spatial arrangement of proteins in planar and curved membranes by PPM 3.0. *Protein Sci.* **31**, 209–220 (2022).
7. Lu, C. *et al.* OPLS4: Improving force field accuracy on challenging regimes of chemical space. *J. Chem. Theory Comput.* **17**, 4291–4300 (2021).
8. Bowers, K. J. *et al.* Scalable algorithms for molecular dynamics simulations on commodity clusters. in *ACM/IEEE SC 2006 Conference (SC'06)* 43–43 (IEEE, 2006). doi:10.1109/sc.2006.54.
